# A new electron diffraction approach for structure refinement applied to Ca_3_Mn_2_O_7_

**DOI:** 10.1107/S2053273321001546

**Published:** 2021-03-17

**Authors:** R. Beanland, K. Smith, P. Vaněk, H. Zhang, A. Hubert, K. Evans, R. A. Römer, S. Kamba

**Affiliations:** a Department of Physics, University of Warwick, Coventry CV4 7AL, United Kingdom; b Institute of Physics, Academy of Sciences of the Czech Republic, Na Slovance 2, 182 21 Prague 8, Czech Republic; c Material Measurement Laboratory, National Institute of Standards and Technology, 100 Bureau Drive, Gaithersburg, MD 20899, USA

**Keywords:** digital diffraction, electron diffraction, Ca_3_Mn_2_O_7_, CBED, LACBED

## Abstract

The digital large-angle convergent-beam electron diffraction (D-LACBED) technique is applied to Ca_3_Mn_2_O_7_ for a range of temperatures. Bloch-wave simulations are used to examine the effects that changes in different parameters have on the intensity in D-LACBED patterns, and atomic coordinates, thermal atomic displacement parameters and apparent occupancy are refined to achieve a good fit between simulation and experiment. The sensitivity of the technique to subtle changes in structure is demonstrated. Refined structures are in good agreement with previous determinations of Ca_3_Mn_2_O_7_ and show the decay of anti-phase oxygen octahedral tilts perpendicular to the *c* axis of the *A*2_1_ *am* unit cell with increasing temperature, as well as the robustness of oxygen octahedral tilts about the *c* axis up to ∼400°C. The technique samples only the zero-order Laue zone and is therefore insensitive to atom displacements along the electron-beam direction. For this reason it is not possible to distinguish between in-phase and anti-phase oxygen octahedral tilting about the *c* axis using the [110] data collected in this study.

## Abstract 2

The ‘digital’ large-angle convergent-beam electron diffraction (D-LACBED) method uses computer control of a transmission electron microscope to collect hundreds of diffraction patterns from a region a few nanometres in size, which are combined into a single data set. The sensitivity of the resulting patterns to crystal structure is explored using the Ruddlesden–Popper oxide Ca_3_Mn_2_O_7_ and it is found that refinement of atomic coordinates can be performed to sub-picometre precision.

## 1. Introduction   

Convergent-beam electron diffraction (CBED) has a long history of application to symmetry determination (Buxton *et al.*, 1976 ▸) and the accurate measurement of individual structure factors (Spence *et al.*, 1989 ▸; Zuo, 1998 ▸; Zuo & Spence, 2017 ▸). However, despite its demonstrated sensitivity to many interesting and useful parameters, it is rarely used for structural refinement (Ogata *et al.*, 2004 ▸). Recently, we have demonstrated that atomic coordinates can be extracted from ‘digital’ large-angle convergent-beam electron diffraction (D-LACBED) patterns (Beanland *et al.*, 2013 ▸) with sub-picometre precision and accuracy using Al_2_O_3_ as a model material (Hubert *et al.*, 2019 ▸). A similar data-collection approach was also developed by Koch with an additional post-specimen de-scan, known as LARBED (Koch, 2011 ▸). D-LACBED patterns, formed by combining hundreds or thousands of CBED patterns each with a slightly different incident-beam orientation, have the advantage of a much larger angular range and many more data points than conventional CBED. They also can be obtained from a region the size of the convergent beam and a single measurement accesses many diffracted beams, unlike conventional LACBED (Tanaka *et al.*, 1980 ▸). The crucial advantage that accrues from collection of large data sets from a single crystal using electron diffraction is that sufficient data are obtained to allow both structure solution and refinement with high accuracy. New data-collection regimes, mainly using hundreds of selected-area or nanobeam spot diffraction patterns obtained while rotating a crystal over a large angle, have now established electron diffraction as a powerful technique for crystal structure solution (Wolff *et al.*, 2020 ▸; Gemmi *et al.*, 2019 ▸) and refinement to sub-picometre precision (Palatinus *et al.*, 2015*a* ▸,*b* ▸). Here, we investigate large data sets obtained from a single specimen orientation. The interference between different Bloch waves as they propagate through a crystalline specimen produces regions in D-LACBED patterns that are particularly sensitive to small changes in phase and give a unique fingerprint for different parameters. We apply the D-LACBED technique to the Ruddlesden–Popper phase Ca_3_Mn_2_O_7_, which has attracted interest as a multiferroic (Benedek & Fennie, 2011 ▸; Fawcett *et al.*, 1998 ▸) with the emergence of ferroelectric–ferromagnetic coupling occurring through the coupling of two non-polar modes with different symmetries. This effect has become known as hybrid improper ferroelectricity (Benedek & Fennie, 2011 ▸) and is currently being studied in a variety of related materials (Senn *et al.*, 2015 ▸; Bansal *et al.*, 2019 ▸; Xu *et al.*, 2019 ▸; Chen *et al.*, 2020 ▸).

**Figure 1:**
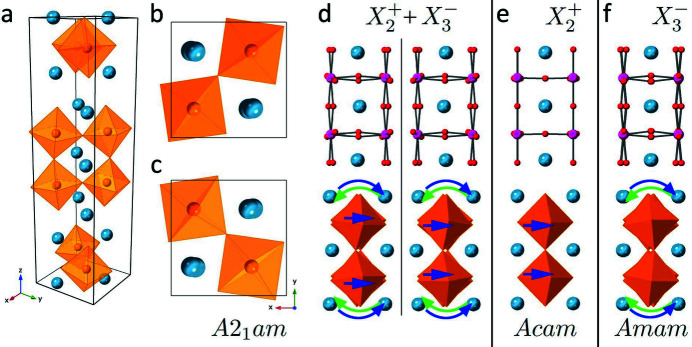
The RT *A*2_1_ *am* structure of Ca_3_Mn_2_O_7_ (Guiblin *et al.*, 2002 ▸) and possible intermediate phases. (*a*) Perspective view of the *A*2_1_ *am* unit cell, Ca atoms blue and MnO_6_ octahedra orange. (*b*) View along [001] showing the upper and lower oxygen octahedra, which twist in-phase about the [001] axis. (*c*) View along [001] showing the central octahedra, which also twist in-phase about [001]. (*d*) Projections of the *A*2_1_ *am* structure, as a ball-and-stick model for the upper perovskite block (Mn atoms magenta, oxygen atoms red, Ca atoms blue) and showing oxygen octahedra for the lower perovskite block. The RT structure has 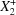 and 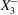 distortions, and appears differently when viewed along [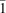
10] (left) and [110] (right). Note the lack of mirror symmetry: the anti-phase octahedral tilts about [110] and in-phase tilts about [001] split the visible oxygen sites and displace the oxygen atoms adjacent to the Mn atom asymmetrically. (*e*) A structure with the 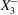 distortion only, with symmetry *Aeam*. (*f*) A structure with the 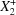 distortion only, with symmetry *Amam*. For both (*e*) and (*f*) the [110] projection is identical to the [1
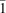
0] projection.

Ruddlesden–Popper phases in general have the formula *A*_*n*+1_ *B*_*n*_O_3*n*+1_ and a structure that consists of a rocksalt *A*O bilayer every *n* *AB*O_3_ perovskite unit cells along [001]. In Ca_3_Mn_2_O_7_ *n* = 2 and the oxygen atoms form corner-sharing MnO_6_ octahedra. The prototype (undistorted) structure has space group *I*4/*mmm* (No. 139) at high temperature. Just as in *AB*O_3_ perovskites, these octahedra can tilt and distort, here lowering the symmetry from the prototype structure and giving rise to macroscopic ferroic behaviour. An orthorhombic structure for La_2−2*x*_Ca_1+2*x*_Mn_2_O_7_ (0.8 < *x* < 1) with space group *Cmc*2_1_ (No. 36) or *Ama2* (No. 40) was proposed by Bendersky *et al.* based on a transmission electron microscopy (TEM) and electron diffraction study (Bendersky *et al.*, 2001 ▸, 2003 ▸). Guiblin *et al*. (2002 ▸) then performed structural refinement at room temperature (RT) using single-crystal X-ray diffraction, only finding an acceptable fit for *Cmc*2_1_. They used the alternative (non-standard) setting *A*2_1_ *am*, which allows the long axis of the unit cell to always be the *c* axis, although there is a ∼45° difference in the *a* and *b* axes between *I*4/*mmm* and *A*2_1_ *am* due to a doubling of periodicity caused by the oxygen octahedral tilting. We use the same setting here, *i.e.* perform refinements in the space group *A*2_1_ *am*. A similar structural determination was obtained by Lobanov *et al.* (2004 ▸) using powder neutron diffraction, which also gave magnetic data, although a minority additional phase was detected at RT, using very high resolution X-ray measurements, that was assigned to be *I*4/*mmm*. The *A*2_1_ *am* RT structure is shown in Figs. 1 ▸(*a*)–1 ▸(*d*). The oxygen octahedra are both anti-phase tilted about the [110] axis and in-phase twisted about the [001] axis, corresponding to the 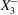 and 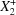 irreducible representations (irreps), respectively (Benedek & Fennie, 2011 ▸). At intermediate temperatures, the evolution of distortion is more complicated. While the 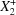 and 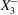 distortions may be coupled at RT, it is less clear that they should have the same behaviour as a function of temperature. In fact, as the material is heated the disappearance of one or other of the distortions before a final transition to the prototype structure might seem inevitable. If the 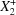 distortion dis­appears leaving only the anti-phase 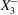 octahedral tilt about [110] [Fig. 1 ▸(*f*)], the structure has space group *Amam* (No. 63). Conversely, the presence of an 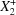 distortion without 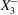 [Fig. 1 ▸(*e*)] produces an *Aeam* (No. 64) structure (Senn *et al.*, 2015 ▸).

However, the study by Senn *et al.*, which examined both Ca_3_Mn_2_O_7_ and Ca_3_Ti_2_O_7_ as a function of temperature using high-resolution X-ray and neutron powder diffraction, gave the unexpected result that *Aeaa* (No. 68) was in fact the best-fit structure to Ca_3_Mn_2_O_7_ at 500 K (227°C). This is surprising because the *Aeaa* space group corresponds to an 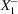 irrep, with anti-phase tilting along the *c* axis. Although *Aeaa* is also a subgroup of the *I*4/*mmm* prototype, it is not an intermediate between *A*2_1_ *am* and *I*4/*mmm*, and can only be in competition with the *Aeam* or *Amam* structures. The transition from *A*2_1_ *am* to *Aeaa* must be of first order, either with or without passing through an intermediate *Aeam* or *Amam* phase. Their data indicated a coexistence of *A*2_1_ *am* and *Aeaa* structures between 200 and 320 K, with single-phase *A*2_1_ *am* below 200 K (−73°C) and pure *Aeaa* above 320 K (∼50°C). This proposal was given further credence by an observed contraction of the *c* axis with increasing temperature, consistent with a ‘trapped’ 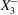 soft phonon mode that can only occur in the *Aeaa* structure, and explained the additional RT phase detected by Lobanov *et al.* (2004 ▸).

Here we consider the question of whether electron diffraction – specifically, D-LACBED – has the accuracy and precision required to provide a refinement of the structure of Ca_3_Mn_2_O_7_. As noted by Senn *et al.*, the *A*2_1_ *am* structure requires 19 free parameters to describe the fractional atomic coordinates of the seven atoms that make up the basis (the unit cell contains 48 atoms in total). In addition, each atom in the basis has an occupancy and thermal atomic displacement parameters and there are the three lattice parameters of the orthorhombic unit cell. We use the term ‘structural parameter’ for all of these variables in what follows. Finally, for electron diffraction four parameters that relate to experiment, rather than the material being examined, also need to be determined – the crystal thickness, the accelerating voltage of the microscope, the angular range covered in the patterns and the point spread function of the camera. Considering only isotropic thermal atomic displacement parameters, this makes 40 parameters in total that could in principle be refined by best match of simulation to experiment for the *A*2_1_ *am* structure. For the *I*4/*mmm* structure, the basis consists of six atoms with only four free parameters describing atomic coordinates, giving 18 structural parameters. While it is obvious that a typical D-LACBED data set, containing >10^7^ pixels, should have sufficient information to refine this comparatively small number of unknown values, whether this is possible in practice is less clear. Furthermore, it is possible that uncontrolled parameters or effects – such as distortions in the experimental patterns, the use of an independent atom model (IAM) for Bloch-wave simulations (Kirkland, 2010 ▸), the lack of any energy filtering or unsophisticated background subtraction – may compromise the measurement and make it unreliable. Finally one must consider the utility of the experiment and the relative difficulty in comparison with X-ray or neutron diffraction: does the D-LACBED method produce information that is different or more easily obtained than that from well established methods?

## 2. Experiment   

Ca_3_Mn_2_O_7_ ceramics were synthesized by solid-state reaction using mechanochemical activation before calcination. CaCO_3_ and MnO_2_ powders (Sigma–Aldrich, 99% purity) were mixed in stoichiometric ratio, then milled intensively in a Fritsch Pulverisette 7 planetary ball micromill for 135 min in a dry environment followed by 23 min in suspension with *n*-heptane. ZrO_2_ grinding bowls (25 ml) and balls (12 mm diameter, acceleration 14*g*) were used. The suspension was dried under an infrared lamp and the dried powder was pressed in a uniaxial press (330 MPa, 3 min) into pellets of diameter 13 mm. The pellets were calcined for 24 h at 1200°C in pure O_2_ to prevent the reduction of Mn4^+^, then milled and pressed by the same procedure as above and sintered at 1300°C for 24 h in pure O_2_. The composition of calcined pellets from X-ray diffraction (XRD) was 97% of Ca_3_Mn_2_O_7_ and 3% of Ca_2_MnO_4_. Traces of Ca_2_MnO_4_ were also observed in sintered pellets. The pellets were then ground and flattened using a dimpler before being ion milled to electron transparency using Ar^+^ ions at 6 kV. The resulting specimens were examined in a standard JEOL 2100 LaB_6_ transmission electron microscope operating at 200 kV.

RT in our TEM setup was measured as 29°C. D-LACBED data were constructed from 3721 CBED patterns using an incident-beam convergence semi-angle of 0.56 mrad and a probe size of approximately 8 nm full width at half-maximum (FWHM) on the specimen. A Ca_3_Mn_2_O_7_ crystal was aligned to the [110] *A*2_1_ *am* zone axis. Patterns were imaged using a Gatan Orius SC600 camera with binning 4. An angular raster of 61 × 61 incident-beam orientations at a rate of approximately 10 patterns s^−1^ covered an angular range of 35 mrad. Data were collected from roughly the same area at nominal temperatures of 100, 200, 300 and 400°C with heating provided by a Gatan 652 double-tilt heating holder. Due to the distance between the electron-transparent area and the heating element, actual temperatures are likely to be slightly lower than nominal ones. Irreversible changes were observed in the specimen at nominal temperatures of 500°C and higher, presumably due to loss of oxygen; these results were discarded as being unreliable. For temperatures above 100°C only 2601 (51 × 51) CBED patterns were collected covering 29.3 mrad, since the sample was slightly less stable and subject to drift of more than a few nanometres. Diffuse background was subtracted from the CBED discs by 2D cubic spline interpolation. Patterns were rotated to give *h*00 horizontal using bicubic interpolation and cropped to 400 × 400 pixels, reducing the angular range to 20 mrad. Where symmetry was evident, averaging was used to improve the signal-to-noise ratio (*i.e.* taking the mean of equivalent patterns, sub-pixel aligned using cross-correlation) and symmetry was also used to correct linear distortions due to intermediate lens aberrations. Linear image distortions that could not be corrected this way were removed by comparison with simulations.

Refinement of parameters to optimize the fit between experiment and simulation was performed using the Bloch-wave simulation program *felix* and the source code is freely available (Beanland *et al.*, 2019 ▸). Here we refer to simulations as LACBED patterns, while retaining the term D-LACBED pattern for experimental data. Experiment and simulation were compared using a zero-mean normalized cross-correlation fit index *Z* for pixel intensities *I*_*p*, *n*_ in LACBED patterns with mean intensities 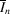 and standard deviation σ_*n*_:
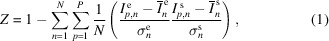
where the sum *p* is performed over all *P* pixels in each pattern and the sum *n* is performed over all *N* experimental D-LACBED patterns. Superscripts e and s refer to experimental and simulated patterns, respectively. This comparison ignores the absolute intensity of each LACBED pattern and concentrates instead on the changes in the detail of each pattern individually. Equation (1) gives a value of zero for a perfect fit and a value of unity for completely uncorrelated experiment and simulation. Data were binned by 4 to 100 × 100 to reduce simulation times and symmetry-related patterns were not included to reduce memory utilization. The simulation uses isotropic Debye–Waller factors and atomic Born scattering factors for neutral atoms from Kirkland (2010 ▸). Absorption is calculated using the numerical integration package *QUADPACK* (Piessens *et al.*, 1983 ▸) following the Bird and King model (Bird & King, 1990 ▸), also using Kirkland scattering factors. Simulations on a cluster used 400 zero-order Laue-zone reflections chosen on a pixel-by-pixel basis from a pool of 1200, which typically completed in 108 s using 168 cores. Changes in intensity in the set of LACBED patterns produced by any structural parameter are both complex and distinctive, which allows a best fit to experimental D-LACBED patterns to be optimized for each parameter independently using a simple downhill gradient optimization routine (bisection + parabolic fit). The same solution was always obtained within estimated error, independent of starting position, indicating the absence of local minima that would give incorrect solutions. Specimen thicknesses were matched to simulation with a precision of 1 nm, using the ability of Bloch-wave methods to simultaneously calculate LACBED patterns for multiple thicknesses and choosing the best fit (see Table 1 ▸ and the supporting information). Unlike refinements using spot patterns (*e.g.* Jansen *et al.*, 1998 ▸), in which almost equivalently good solutions can be obtained for different thicknesses, the large number of patterns in D-LACBED data results in a single best fit (Hubert *et al.*, 2019 ▸). In order to estimate errors, after a RT best-fit structure was obtained we determined the standard deviation of individual atomic coordinates obtained from a multitude of starting points. These errors scale in the same way as the relative sensitivity of D-LACBED data to different parameters (see Section 3.4). They are roughly equal to the change in structural parameter that produces an increase of 10^−5^ in *Z* (see Fig. S1 in the supporting information). Thus, to avoid the need for multiple simulations to estimate errors, the latter was used as a consistent and readily calculated proxy.

## 3. Results   

### 3.1. RT imaging and diffraction   

**Figure 2:**
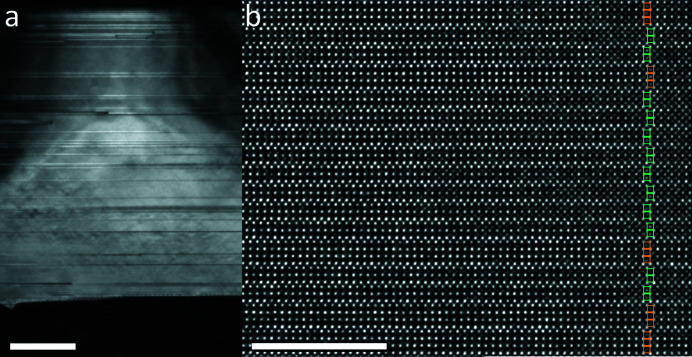
Transmission electron micrographs of Ca_3_Mn_2_O_7_ at the [110] *A*2_1_ *am* zone axis. (*a*) Dark-field 2
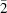
0 image showing planar defects and contrast bands in a single grain (scale bar 200 nm). (*b*) High-resolution [110] lattice image showing the occurrence of *n* = 3 perovskite blocks forming planar defects (scale bar 10 nm).

A typical TEM image at RT is shown in Fig. 2 ▸(*a*). Grains, often with (001) planar boundaries, were up to 2 µm in size. Imaging perpendicular to the [001] direction reveals a significant density of (001) stacking faults, with a spacing between 2 and 100 nm, *i.e.* between 1 and 50 unit cells. These faults are interruptions in the 2:1 perovskite:rocksalt *n* = 2 Ruddlesden–Popper stacking sequence and were found to always consist of additional perovskite layers, indicating a slight excess (∼1% Mn) above the nominal 2:3 Mn:Ca ratio. Bands of brighter and darker contrast are observed in dark-field images as can be seen in the upper part of Fig. 2 ▸(*a*). As will be shown later, these bands are merohedral twins, as first proposed by Bendersky *et al.* (2003 ▸) and also observed by Gao *et al.* (2017 ▸). D-LACBED data were collected from the centre of stacking-fault-free regions, typically between 30 and 100 nm in size, using an electron probe of 10 nm FWHM.

### 3.2. RT D-LACBED   

**Figure 3:**
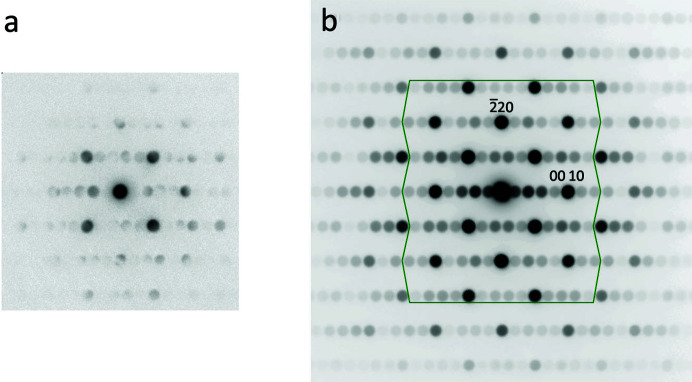
(*a*) Individual RT CBED pattern obtained at the Ca_3_Mn_2_O_7_ [110] *A*2_1_ *am* zone axis. (*b*) Average RT CBED pattern, the sum of 3721 CBED patterns. The 109 spots used to reconstruct D-LACBED patterns lie inside the green boundary.

Fig. 3 ▸(*a*) shows an individual RT Ca_3_Mn_2_O_7_ CBED pattern at an incident-beam orientation very close to the [110] zone axis, while Fig. 3 ▸(*b*) shows the average CBED pattern, obtained by aligning the 000 beam of all 3721 CBED patterns prior to summation. Due to the relatively large angular range covered by the incident beam, the average CBED pattern shows a more even distribution of intensities and extends further out in reciprocal space, a similar effect to that seen in precession electron diffraction (PED) (Vincent & Midgley, 1994 ▸). Here, we only reconstruct D-LACBED patterns that have their pattern centre within the angular range covered, and choose the 109 discs inside the marked area in Fig. 3 ▸(*b*).

**Figure 4:**
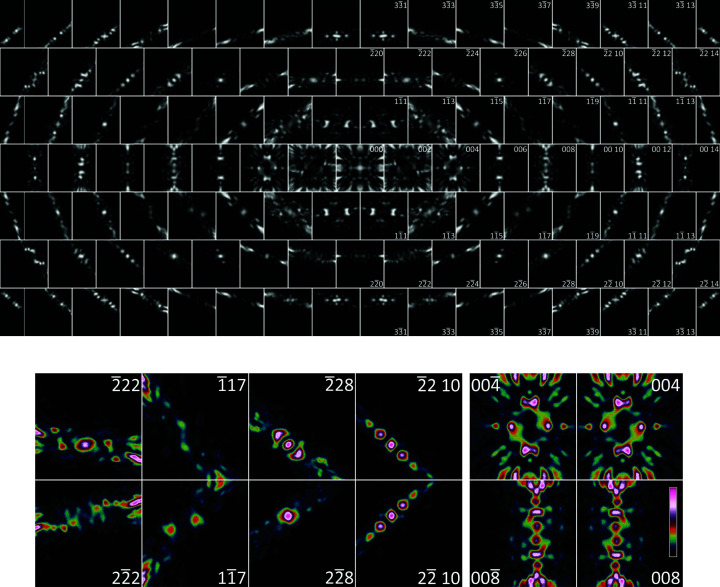
(Top) The 109 experimental RT (29°C) D-LACBED patterns at the Ca_3_Mn_2_O_7_ [110] zone axis corresponding to the spots indicated in Fig. 3 ▸(*b*). All patterns have an angular range of 20 mrad and are normalized to the full intensity range for display. A mirror is present on (001) but not (1
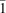
0), which can be seen in 00*h* patterns and by comparing Bijvoet pairs 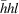 and 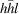 (false colour patterns, bottom).

The corresponding RT D-LACBED patterns are shown in Fig. 4 ▸. As we have noted before (Beanland *et al.*, 2013 ▸), determination of symmetry using D-LACBED patterns is straightforward in comparison with other methods, particularly for materials with a large lattice parameter that restricts the convergence angle of the incident beam. In the current case, the assembly of D-LACBED patterns in the upper part of Fig. 4 ▸ exhibits a (001) mirror, but not a (1
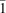
0) mirror, as expected from the [110] projection of the RT structure in Fig. 1 ▸(*d*). Since electron diffraction does not obey Friedel’s law, the lack of a centre of symmetry is visible from the difference between 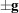 patterns, or equivalently by comparison of Bijvoet pairs 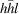 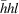 related by the broken mirror, as shown for selected patterns in the lower part of Fig. 4 ▸. The D-LACBED patterns on the left show that symmetry breaking is quite striking in some cases (*e.g.* 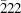-, 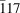-, 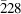-type patterns), but hardly visible in others (*e.g.* 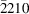-type patterns). The presence of (001) mirror symmetry and absence of (110) mirror symmetry is also visible in the D-LACBED patterns on the 00*h* systematic row. As is clear from Fig. 1 ▸(*d*), the symmetry is broken primarily by oxygen-atom displacements of a few picometres, demonstrating the sensitivity of D-LACBED to the coordinates of relatively light elements. It is impossible to observe the symmetry in the conventional CBED pattern [Fig. 3 ▸(*a*)] – in fact, since the discs must be so small to avoid overlap and are essentially featureless, it is hardly possible to align the crystal exactly on the zone axis. Furthermore, while the average CBED pattern contains significant intensities that cover a larger angular range [Fig. 3 ▸(*b*)], the absence of the (1
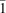
0) mirror is still not evident, presumably because the intensity differences only affect a small part of the the D-LACBED patterns and so have a small impact on the total intensity. We expect this problem may also affect PED data, which may not sample the particular incident-beam orientations that produce strong intensity differences between Bijvoet pairs.

**Figure 5:**
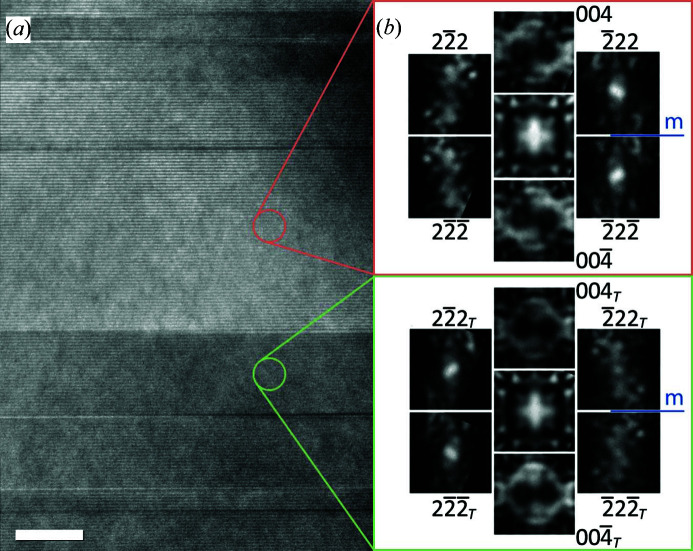
(*a*) Contrast bands in a bright-field TEM image of Ca_3_Mn_2_O_7_ close to the [110] axis. Scale bar 20 nm. (*b*) Selected D-LACBED patterns taken from different bands showing that the structures are related by a twinning operation such as a (1
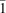
0) mirror or an inversion, although it is not possible to distinguish between them.

The difference in 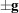 D-LACBED patterns in Fig. 4 ▸ provides a simple method to demonstrate that the dark and light bands seen in dark-field TEM images are caused by merohedral twinning. Four twin variants are expected from the ratio of the order of the point groups of the prototype and RT structures, 4/*mmm* (order 16) and *mm*2 (order 4), respectively, *i.e.* 4/*mmm* = *mm*2 × 2/*m*, where × indicates a direct group product. In the case of *A*2_1_ *am* Ca_3_Mn_2_O_7_, one may choose 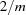 = 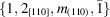. However, since D-LACBED patterns are from the zero-order Laue zone (ZOLZ) they do not contain any 3D information from higher-order Laue zones (HOLZ). They correspond to a projection of the crystal structure and it is not possible to distinguish between all four of these different twinning operations; a twin generated by 2_[110]_ is invisible and that generated by *m*_(110)_ appears identical to that generated by 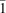. This explains the observation of only two types of contrast, dark and light, even though there are four distinct twin variants. Fig. 5 ▸ shows selected D-LACBED patterns, 004- and 222-type, which are sensitive to the broken mirror symmetry obtained from bands of different contrast. Reflections that appear equivalent in conventional [CBED, selected-area electron diffraction (SAED)] diffraction patterns are labelled with the same indices, using the subscript *T* to denote twinned (darker) material. The relationship between the different bands is readily apparent, *e.g.* the 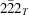 pattern from the dark band is equivalent to the 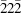 pattern from the light band.

### 3.3. Structural refinement   

We first consider atomic coordinates, with 19 structural parameters. Refinement was performed by minimizing differences between simulations and experimental RT data as described in Section 2, starting from the *I*4/*mmm* structure given by Bendersky *et al.* (2003 ▸) in the space group *A*2_1_ *am* with some small initial displacements from *I*4/*mmm* to ensure that the sense of octahedral rotations was consistent with the model of Lobanov *et al.* (2004 ▸). The initial fit was *R* ∼ 10%, which refined to *Z* = 3.7%. The refined RT structure is given in Table 1 ▸. Column δ*s* gives the difference in picometres between this refinement and the structure determined by Lobanov *et al.* using synchrotron X-ray diffraction. Average disagreement for all coordinates is 1.7 pm, with the largest difference (9 pm) in the position of the Ca atoms; our refinement is closer to that of Lobanov *et al.* than is the X-ray study of Guiblin *et al.* (2002 ▸). This result clearly demonstrates the ability of D-LACBED to reliably determine structure with a precision comparable with that of other diffraction methods. Differences in electron and X-ray/neutron refinements may be due to the inevitable inclusion of stacking faults and small amounts of other phases in the X-ray/neutron data (Lobanov *et al.*, 2004 ▸), which are avoided by the local measurement in defect-free material that is possible with D-LACBED. The RT structure refined from our D-LACBED data shows octahedral tilting about *x* and *z* axes, as expected.

Second, D-LACBED data are also sensitive to thermal atomic displacement factors *B* and apparent occupancy. The influence of *B* on D-LACBED patterns is typically ∼50 times less than that of atomic coordinates. Optimizing the fit to experiment improved *Z* by at most 0.5% and had no noticeable effect on atomic coordinate refinement. The lower sensitivity is reflected in the lower precision of *B* factors in Table 1 ▸. Our previous study (Hubert *et al.*, 2019 ▸) found that thermal atomic displacement parameters in GaAs were significantly overestimated in D-LACBED refinements. This also is the case here: Guiblin *et al.* give *B*_Ca_ = 0.58 Å^2^, *B*_Mn_ = 0.30 Å^2^ and *B*_O_ ≃ 0.65 Å^2^ at RT. Since the main effect of thermal atomic vibrations on LACBED patterns is to change absorption, the discrepancy may indicate that an improvement in the subtraction of diffuse background intensity in the original CBED patterns is required. Nevertheless, as shown in Section 3.5, *B* factors determined from D-LACBED data increase with temperature, as expected. An additional improvement in *Z* of at most 0.5% is obtained by allowing occupancy to vary, shown in the final column of Table 1 ▸. Oxygen stoichiometry may be affected by, for example, loss of oxygen under the electron beam and/or during heating in the vacuum of the microscope. However the best fit for oxygen occupancy at RT, between 0.9 and 0.95, suggests a very large oxygen loss that does not seem reasonable. This discrepancy is almost certainly due to the neutral IAM used here for structure factors; a lower measurement of occupancy corresponds to a shallower potential, consistent with the O^2−^ anions in this material. This explanation is supported by occupancies very slightly in excess of unity for both Ca and Mn cations. We observe similar effects consistent with charge transfer between cations and anions in all other oxides studied by D-LACBED using a neutral IAM. Bonding and charge transfer principally affect low-order structure factors (Zuo, 1998 ▸) and have been targeted in many quantitative CBED studies (*e.g.* Tsuda & Tanaka, 1995 ▸; Nakashima *et al.*, 2011 ▸) as well as least-squares refinement of structure factors using multislice simulations (Jansen *et al.*, 1998 ▸). The effects of changes to individual structure-factor amplitudes or phases are distributed across all patterns due to multiple (dynamical) scattering in a similar manner to structural parameters as investigated below in Section 3.4.

Finally, D-LACBED data are also sensitive to the lattice parameter, but unlike the other structural refinements this changes the position of the Bragg condition in the pattern as well as changes intensities from place to place. Our cross-correlation equation (1) requires both the angular range of the D-LACBED pattern to be known, and the position of the Bragg condition within it to be aligned to that of the simulated LACBED pattern with sub-pixel precision. We find that the presence of distortions such as intermediate lens astigmatism prevents a precise and absolute calibration of angular range to better than 0.5%, which is vastly inferior to X-ray measurements and not sufficient to allow reliable refinement of the lattice parameter using D-LACBED. We thus use values from other studies (Senn *et al.*, 2015 ▸).

### 3.4. Sensitivity of D-LACBED data   

**Figure 6:**
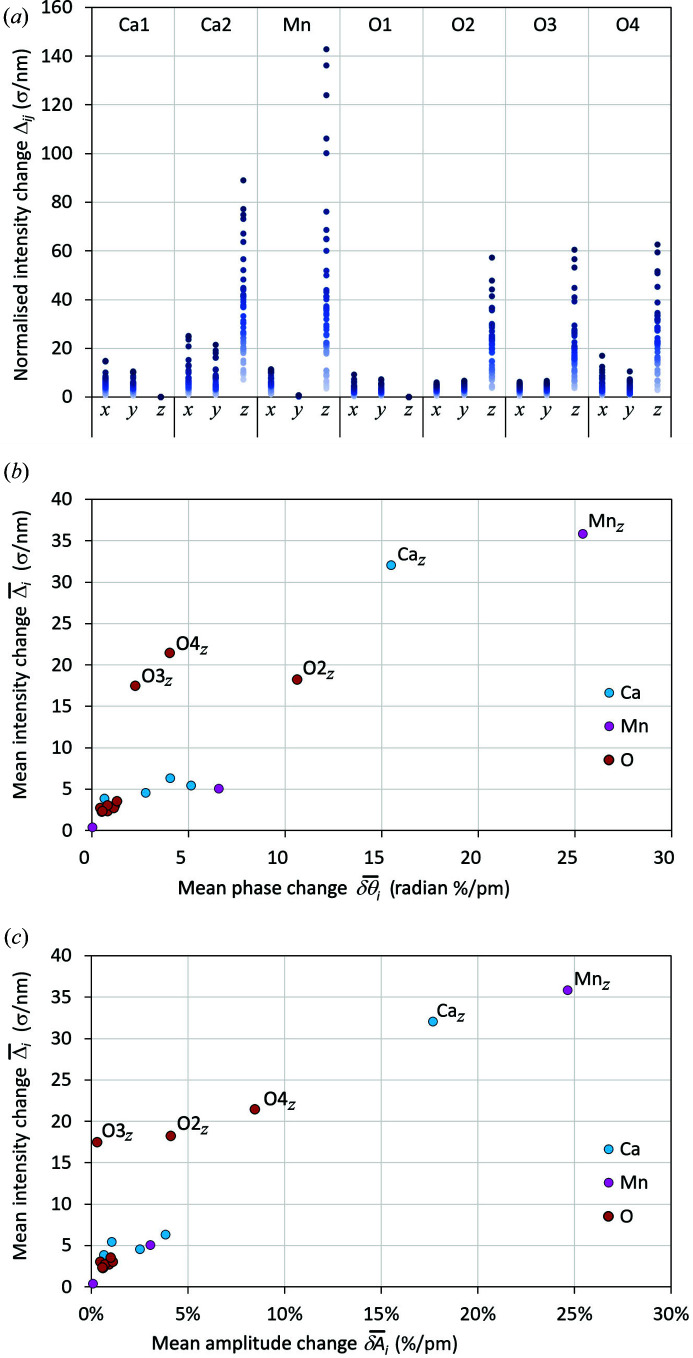
(*a*) Normalized r.m.s. changes in intensity Δ_*ij*_ of the 54 unique [110] LACBED patterns for the 19 allowed atom displacements within the constraints of *A*2_1_ *am* (note Ca1 *z* and O1 *z* are fixed at zero). The change in intensity is given in units of standard deviation of the image per nanometre movement. (*b*) The correlation between the average of all LACBED intensity changes 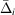 and the average change in structure-factor phase 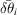 for the 19 allowed atom displacements. (*c*) The same correlation for the mean change in structure-factor amplitude 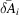.

The structure given in Table 1 ▸ clearly shows that structural refinement from D-LACBED data is viable even for quite complex inorganic materials. We now consider the sensitivity of LACBED patterns to the different structural parameters; we find that they depend strongly upon which atom, and which coordinate, is being refined. The effect of a structural parameter can be quantified by taking the difference between zero-mean normalized LACBED patterns for a reference structure *S* and an altered one *S*′, in which a small change in the *i*th structural parameter is made, in the current case a small change in atomic coordinate δ*s*_*i*_. The result is an image that has positive and negative values corresponding to increases or decreases in intensity produced by the small change in structural parameter, which we call a δ image. Dividing by the magnitude of the displacement allows a direct comparison between different coordinates, 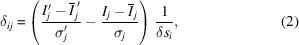
where σ_*j*_ is the standard deviation of the pixel intensities in pattern *j*. The δ images are shown for all 54 patterns *j* and all 19 allowed atomic displacements *i* for *A*2_1_ *am* Ca_3_Mn_2_O_7_ in the supporting information. We may assess the relative effect of each structural parameter by taking the root mean square (r.m.s.) of the δ image, *i.e.*

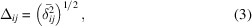
plotted in Fig. 6 ▸. A larger Δ_*ij*_ corresponds to a larger change in the intensities of LACBED pattern *j*. Thus, in Fig. 6 ▸(*a*), each point corresponds to an individual Δ_*ij*_ for the refined RT structure, ordered by the structural parameter *i*. A similar plot can be used to assess the goodness of fit between simulation and experiment, as shown in Fig. S3, which shows the general improvement in fit for all patterns as the structure is refined from *I*4/*mmm* to *A*2_1_ *am*, but also the improvement in the fit for specific patterns such as 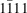 and 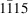. The different atomic coordinates have differing effects on the LACBED patterns, and it is instructive to examine this in more detail. An obvious dependence on atomic number is present, with the largest changes produced by the *z* coordinate of Mn (*Z* = 25), followed by Ca (*Z* = 20) and oxygen (*Z* = 8). The ratio of maximum intensity changes produced by equal movements of the three elements, *I*_max_(Mn):*I*_max_(Ca):*I*_max_(O), is 7.0:4.3:1, which indicates a dependence of roughly *Z* ^1.6^. A second clear trend is the dependence of Δ_*ij*_ on the ordinate being refined: *z* coordinates have roughly 4 to 10 times larger influence on the patterns than *y* and *x* coordinates. This can be partly understood by noting that the *z* coordinate is perpendicular to the incident-beam direction; movements along *z* lie completely in the [110] projection, whereas movements along both *x* and *y* are close to 45° to the beam direction and so produce displacements in this projection that are smaller by a factor of cos(45°). However, the *y* coordinate of Mn produces the smallest intensity changes of all, which seems to be in contradiction with the dependence upon atomic number noted above.

Further insight may be gained into the the sensitivity of LACBED patterns to changes in atomic coordinates by considering the corresponding structure factors. The change in the structure factor of the *j*th reflection *F*_*j*_ due to a change in the *i*th structural parameter is 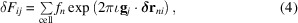
where the sum is performed over all *n* atoms in the unit cell, *f*_*n*_ is the scattering factor of the *n*th atom and ι = (−1)^1/2^. We find that plotting δ*F*_*ij*_ against Δ_*ij*_ yields no correlation (plots showing δ*F*_*ij*_ for all 19 coordinate parameters in *A*2_1_ *am* Ca_3_Mn_2_O_7_ are shown in the supporting information). This lack of correlation is due to the transfer of intensity between different **g** vectors due to dynamical scattering; notably, large intensity changes occur even for LACBED patterns that have precisely zero change in amplitude or phase. However, for a given structural change *i* there is a positive correlation between the *average* change in structure factor of all reflections 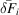 and the *average* of all LACBED intensity changes 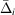, shown in Figs. 6 ▸(*b*) and 6 ▸(*c*) in terms of mean change in structure-factor amplitude 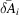 and phase 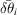.

In summary, structural parameters that have large effects on structure factors do produce large changes in the intensity of LACBED patterns, but these intensity changes are redistributed through the set of patterns by dynamical scattering. Equally, changes that have little effect on structure factors have a lesser effect on LACBED patterns. Although the magnitude of a change in intensity in any individual pattern may effectively be uncorrelated to that of the underlying cause, the complete set of LACBED patterns shows a similar overall sensitivity to that expected from kinematical scattering. This explains the relative lack of sensitivity of the D-LACBED data to the Mn *x* coordinate, which produces only very small changes in the structure factors of the LACBED patterns at the [110] zone axis [the point close to the origin in Figs. 6 ▸(*b*) and 6 ▸(*c*)].

**Figure 7:**
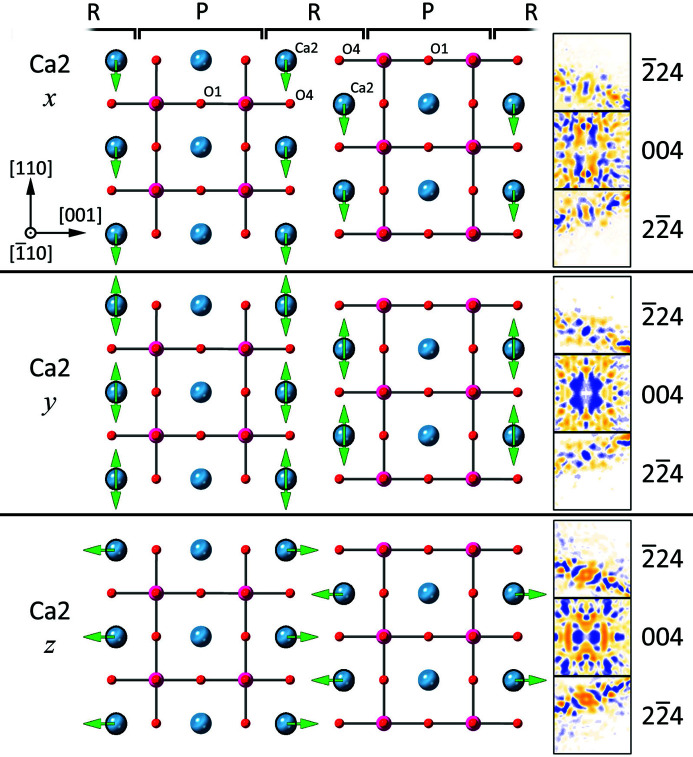
(Left) The movements of atom Ca2 from the prototype structure within space group *A*2_1_ *am* when displaced along the *x*, *y* and *z* directions, as seen in the [110] projection. Colour coding is Ca blue, Mn magenta and oxygen red; perovskite/rocksalt layers are labelled P and R, respectively. There are eight symmetrically equivalent Ca2 atoms in the unit cell, which lie in the rocksalt layers, while Ca1 atoms lie in the perovskite layers. (Right) Three δ_*ij*_ patterns (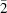
24, 004, 2
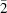
4) showing the resulting changes in intensity (yellow = positive, blue = negative). Displacement of Ca2 along *x* breaks mirror symmetry and produces antisymmetric δ_*ij*_ patterns. Displacement of Ca2 along *y* moves atoms at different depths in opposite directions, maintaining mirror symmetry in the projection and δ_*ij*_ patterns, while displacement along *z* moves Ca2 away from the perovskite layers, also maintaining mirror symmetry. See also the video and Fig. S4 in the supporting information.

We now understand that the average magnitudes of intensity changes 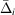 are correlated with the average change in structure factor, but have not considered why these should be so different for the particular case of Ca_3_Mn_2_O_7_. Certainly the dependence on scattering factor implicit in the structure-factor equation (4) explains the effect of atomic number, but why is there such a difference between different ordinates? We may understand this more clearly by examining the allowed displacements within the *A*2_1_ *am* space group, as shown for the Ca2 atom in Fig. 7 ▸. The Ca2 atom lies in the rocksalt layer of the Ruddlesden–Popper structure with a multiplicity of eight in the unit cell. Displacing a Ca2 atom along *x* results in a movement of all Ca2 atoms in the same direction when viewed in the [110] projection as shown in Fig. 6 ▸(*a*). Conversely, a displacement along *y* moves atoms at different depths in opposite directions, and what appears as a single atom column in *I*4/*mmm* splits into a pair of overlapping atoms at different depths. Finally, displacement along *z* moves the Ca2 atoms further towards the centre of the rocksalt layers. Movements of the other atoms are similar, as illustrated in the video in the supporting information. The very small Δ_*ij*_ values for *y* movements of the Mn atom may be caused by two factors: first, the centroid of the atom column remains unchanged and a *y* displacement therefore does not change the phase of low-order structure factors; and second the close and symmetrically arranged O2 and O3 atoms overlap the Mn atom completely. The latter probably indicates that D-LACBED refinement loses some precision when atoms overlap in projection and may be a limiting factor in the technique.

In Fig. 7 ▸ the atoms are displaced from the tetragonal *I*4/*mmm* structure. The effect on the symmetry of the whole LACBED data set is illustrated using a triplet of δ images (the 004 pattern and the Bijvoet pair 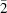
24, 2
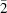
4) on the right. Displacements along *y* and *z* do not break mirror symmetry in the [110] projection and thus the δ patterns maintain this symmetry. Conversely, a shift along *x* does break mirror symmetry and the δ patterns are antisymmetric, with opposite changes in intensity for Bijvoet pairs. The presence of twofold symmetry in all LACBED patterns produces lines of zero intensity change in the systematic 001 row in the latter case, a similar effect to the well known Gjonnes–Moodie bars but in this case in the differential of the LACBED pattern δ_*i*_. More complicated changes can be produced by displacement of the oxygen atoms, for example movements of atoms O2 and O3 have δ images without mirror or antimirror symmetry. However, mirror symmetry can be maintained by coupled movement of the oxygen atoms, for example when O2*z* and O3*z* change by equal amounts in the same direction, or when O2*x* and O3*x* change by equal amounts in opposite directions (see Fig. S4).

In summary, while it is operationally straightforward to refine structural parameters using a simple fit parameter and a downhill gradient optimization, the sensitivity of the technique depends on the details of the structure being examined. The symmetry visible in D-LACBED patterns provides extra information, which can be used to constrain the permissible structural distortions in a structure. We make use of this additional constraint in the following section.

### 3.5. Refinements at elevated temperature   

**Figure 8:**
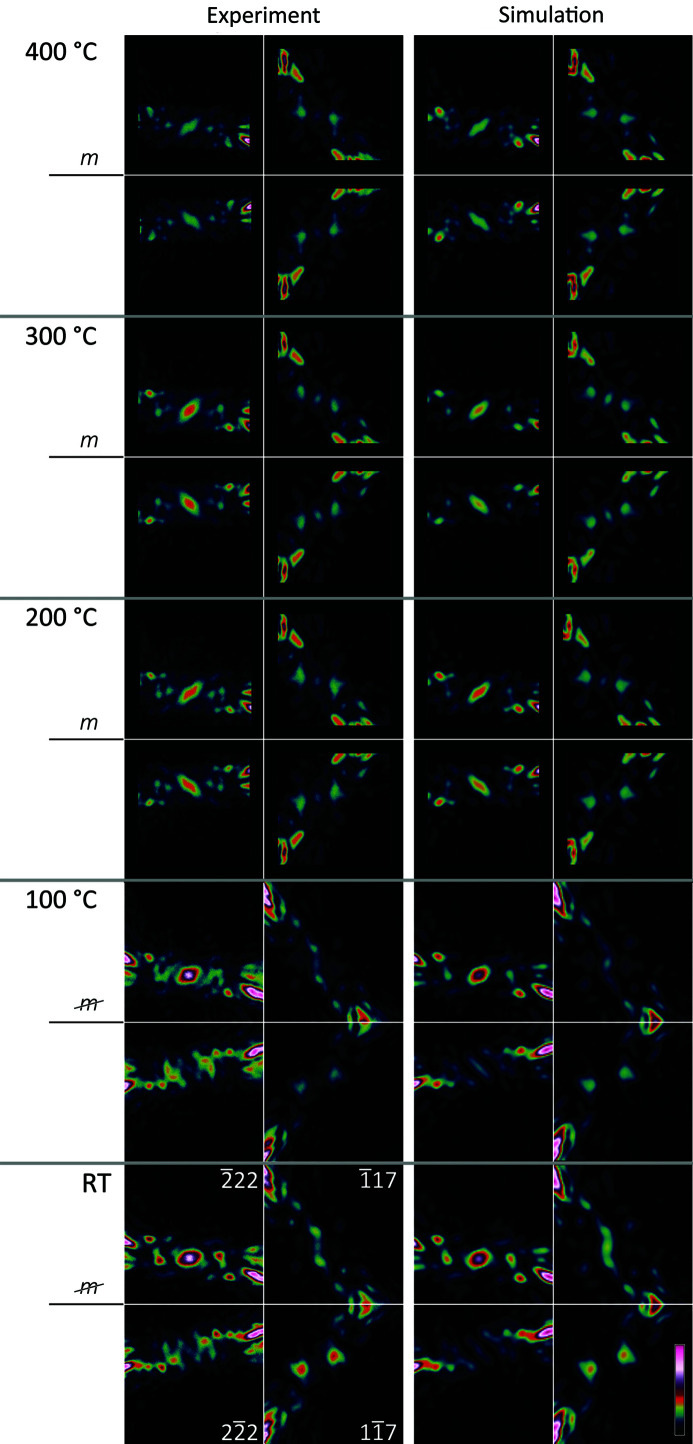
Comparison of experimental D-LACBED patterns (left) and best-fit simulated LACBED patterns (right) at a range of temperatures. The 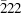 and 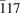 Bijvoet pairs are strongly asymmetric at RT due to the presence of both 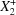 and 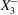 distortions (see Fig. 1 ▸). Mirror symmetry is present at 200°C and higher.

Measurements were taken and refinements performed for nominal sample temperatures up to 400°C, with the starting point for each refinement the best fit determined for the temperature below. Refinements are constrained to the *A*2_1_ *am* space group for all temperatures. [While Senn *et al.* (2015 ▸) proposed the space group *Aeaa* at temperatures where the anti-phase 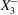 distortion is absent, this gave identical fits since in-phase and anti-phase octahedral twists about [001] appear exactly the same in the [110] projection of these structures. For simplicity here we ignore the possibility of an anti-phase 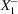 distortion and the *Aeaa* phase on the understanding that the results are also compatible with it.] Selected D-LACBED patterns (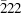, 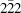 and 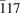, 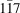) are shown in Fig. 8 ▸ on the left, with best-fit simulations on the right. A good fit is obtained for all cases and the change in symmetry is readily apparent, with the development of (1
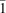
0) mirror symmetry essentially complete by 200°C, indicating that the 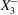 distortion has dropped to zero.

**Figure 9:**
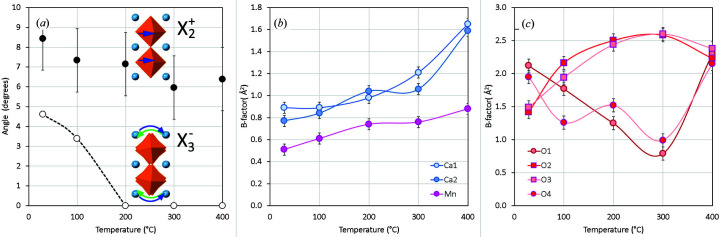
(*a*) The magnitude of oxygen octahedral rotations corresponding to the distortion modes 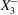 and 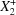 (or 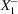). (*b*) Thermal atomic displacement parameters (*B* factors) for Ca and Mn as a function of temperature. (*c*) *B* factors for oxygen atoms showing an anomalous decrease for O1 and O4 up to 300°C.

In refinements at 200°C or higher, mirror symmetry was preserved in the simulations by coupling the movement of oxygen atoms in octahedral rotations about the long *c* axis corresponding to the 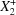 distortion mode, as noted above. Structures are given in the supporting information, Tables S1 to S4. Fig. 9 ▸(*a*) shows the magnitude of the distortions as a function of temperature. Although previous investigations found that tetragonal *I*4/*mmm* symmetry is obtained at temperatures of 400°C and higher, the octahedral rotations about *c* do not drop to zero at 400°C in our measurement and in fact increase between 300 and 400°C. This may be because the temperature of the TEM specimen at the electron probe is rather lower than the nominal value, obtained from a thermo­couple in the specimen holder. The thermal atomic displacement parameters (*B* factors) extracted from D-LACBED data are shown in Figs. 9 ▸(*b*) and 9 ▸(*c*). The Mn and Ca atoms show a monotonic increase in *B* factor with temperature, as expected. Interestingly, oxygen atoms O1 and O4, whose displacements from the sites shown in Fig. 7 ▸ correspond to the 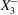 distortion, show a strong decrease in *B* factor up to 300°C, *i.e.* even when the 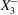 distortion is zero. This may show the presence of strong anharmonicity or rapid hardening of a soft phonon mode above 200°C.

## 4. Discussion   

We begin by addressing the question posed at the end of Section 1, about the information that is produced and the ease of use of the technique in comparison with other methods. The structural measurements made here are quite routine for single-crystal neutron diffraction or synchrotron X-ray diffraction, but have to date not been possible using electron diffraction. While a large amount of data and simulated LACBED patterns are presented here (see the supporting information), this is primarily for the sake of completeness and to explore the possibilities offered by the D-LACBED method, and should not be taken as the level of detail or data processing necessary to obtain a result. In fact, data acquisition is straightforward and takes only a short amount of time, and the level of expertise required is little more than that needed to collect a conventional CBED pattern. Data processing into a form suitable to match simulated patterns typically takes less than an hour. Refinement by matching to Bloch-wave simulations, although robust due to the presence of a global minimum, currently requires access to good computing resources (preferably a cluster with 200 cores or more), but these are widely available and the computation requirement compares favourably with other techniques such as molecular dynamics or density functional theory. Since the technique uses a focused probe that is stationary on the specimen, the electron dose is high (we estimate >10^8^ electrons Å^−2^). Improvements in both experimental and computational methods could easily reduce time and effort by some orders of magnitude, while the relationship between intensities in dynamical diffraction patterns and structural parameters provides an extra dimension to analysis which is available if required. Additionally, refinement of individual structure factors, as typically practised in CBED studies (Zuo, 1998 ▸; Zuo & Spence, 2017 ▸), is manifestly possible using LACBED data. As illustrated here, the D-LACBED technique brings the advantages of TEM, *i.e.* the ability to collect data from nanoscale regions and the opportunity to directly investigate the same region using diffraction contrast or high-resolution imaging, as well as structural determination with sub-nanometre precision. Perhaps the biggest limitation of the technique, at least in its current form where HOLZ information is essentially absent, is the lack of information on atomic displacements parallel to the electron beam. In the case of [110] Ca_3_Mn_2_O_7_ the displacement of one atom parallel to the beam must be accompanied by the displacement of another in a different direction that is related by symmetry, thus ensuring that all atomic coordinates can be refined. This is not the case in general. It may also be the case that atoms that are overlapping in the particular point of view chosen cannot be separated. To get the most from the technique, there may have to be some trade-off between the choice of an incident-beam direction that is sensitive to all atomic displacements while maintaining enough dynamical diffraction to be sensitive to the relative phases of the diffracted beams. Alternatively, refinements may be made using data collected from different zone axes.

In the current refinement these limitations prevent the two models of structure at elevated temperature, *Aeam* and *Aeaa*, from being distinguished, although a repeat experiment at a different zone axis could do so. However, the decay in anti-phase oxygen octahedral tilt (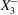) with increasing temperature (Fig. 9 ▸) is quite clear, as is the relative lack of change in octahedral twist about the *c* axis (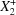). Our results are in good agreement with those of more sophisticated studies such as that of Ye *et al.* (2018 ▸).

## 5. Summary and conclusions   

We have demonstrated that D-LACBED data can be used to determine the relatively subtle structural distortions in Ca_3_Mn_2_O_7_ with a single global minimum in fit parameter that is straightforward to obtain using gradient descent optimization. Refinement of the structural model for RT data in the *A*2_1_ *am* space group gives an excellent fit to simulation. The result agrees with previous X-ray and neutron diffraction measurements to a mean accuracy better than 2 pm. Measurements at elevated temperature confirm the decay in anti-phase oxygen octahedral tilting perpendicular to the *c* axis, producing an *Aeam* or *Aeaa* structure at 200°C and above. The octahedral twists about *c* are robust and persist to a nominal 400°C, in agreement with previous work by others. The high sensitivity of the technique to atomic displacements can be understood by examining the changes in structure factor they produce. While dynamical diffraction effects ensure that changes in intensity for an individual pattern are generally unrelated to the change in its structure factor, taken as a whole an ensemble of D-LACBED patterns demonstrates an average sensitivity that correlates well to the average change of all structure factors. The additional information provided by the symmetry of the D-LACBED patterns allows additional constraints to be applied to a structural model. The main weakness of the technique in its current form is the inability to distinguish between structures that appear the same in projection for the chosen orientation of the crystal. Thermal atomic displacement parameters can also be obtained but are much larger than those obtained by other methods, possibly indicating that improvements in the absorption model or data processing are required. Apparent atomic occupancies may be influenced by charge transfer between atoms, and show the limitations of the neutral IAM used here.

Finally, we note that the method can in principle be used on any modern computer-controlled transmission electron microscope, giving an additional quantitative diffraction method that can be applied at the nanoscale, complementing the already considerable strengths of TEM in imaging and spectroscopy.

## Supplementary Material

Crystal structure: contains datablock(s) 302K, 373K, 473K, 573K, 673K. DOI: 10.1107/S2053273321001546/lu5005sup1.cif

Animation showing constrained atom movements. DOI: 10.1107/S2053273321001546/lu5005sup2.gif

Additional tables and sensitivity plots. DOI: 10.1107/S2053273321001546/lu5005sup3.pdf

CCDC references: 2062211, 2070881, 2070882, 2070883, 2070884

## Figures and Tables

**Table 1:** Refined RT *A*2_1_ *am* structural parameters with a best fit of *Z* = 3.7% at a specimen thickness of 53 nm Column δ*s* gives the difference in picometres between this refinement and the synchrotron X-ray + neutron study of Lobanov *et al*. (2004 ▸). Previous work (Hubert *et al.*, 2019 ▸) indicates that D-LACBED refinements overestimate atomic displacement parameters *B* and that the apparent occupancy is influenced by charge transfer between atoms: here all cations appear to have an occupancy slightly greater than one, and oxygen anions less than one.

Site		Coordinate			δ*s* (pm)	*B* (Å^2^)	Occupancy
Ca1	4*a*	0.7360 (6)	0.2654 (8)	0	[4.2, −9.1, –]	0.91 (5)	1.016 (3)
Ca2	8*b*	0.7578 (4)	0.2657 (5)	0.1861 (1)	[9.0, −6.7, 0.8]	0.79 (5)	1.014 (2)
Mn	8*b*	0.2500 (5)	0.2500 (20)	0.0978 (1)	[−0.8, 1.6, 0.0]	0.52 (4)	1.019 (3)
O1	4*a*	0.2513 (10)	0.2947 (11)	0	[2.5, 2.8, –]	2.2 (1)	0.95 (3)
O2	8*b*	0.0258 (10)	0.5297 (9)	0.1045 (1)	[0.6, 2.1, −0.1]	1.4 (1)	0.91 (1)
O3	8*b*	0.9649 (10)	0.0335 (9)	0.0889 (1)	[−2.0, 2.9, −1.6]	1.5 (1)	0.90 (1)
O4	8*b*	0.2455 (7)	0.2118 (9)	0.1964 (1)	[0.8, 3.9, −2.4]	2.0 (1)	0.96 (1)
