# Supplementary figures and images for: A new electron diffraction approach for structure refinement applied to Ca3Mn2O7
Source: Acta Crystallogr A Found Adv. 2021 Mar 17;77(Pt 3):196–207. doi: 10.1107/S2053273321001546 (PMC8127389; doi:10.1107/S2053273321001546)

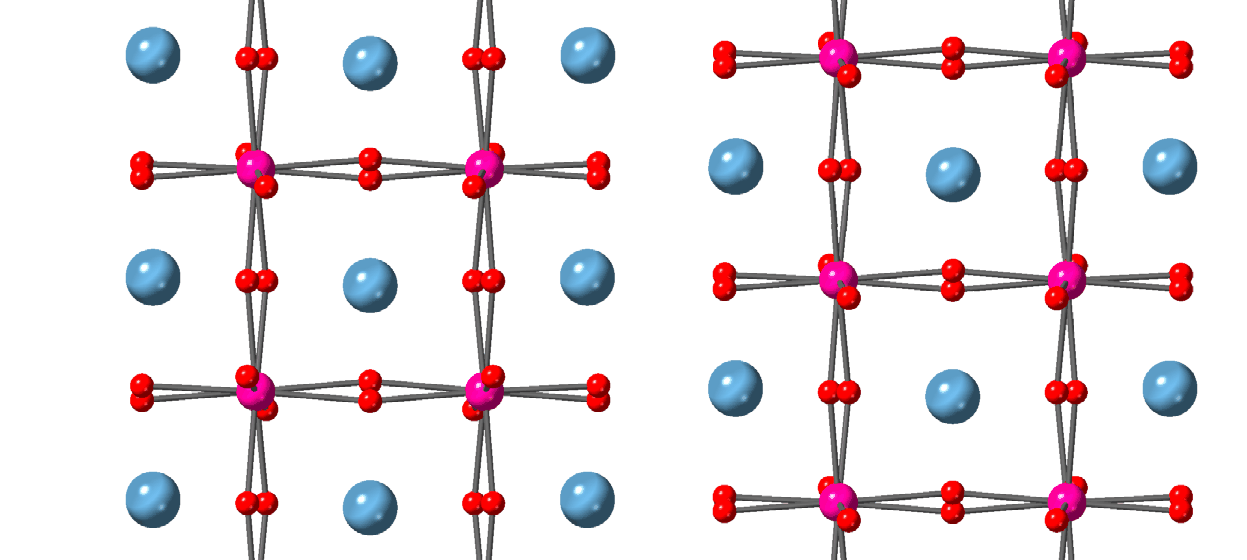

Supplement: Supplementary file 2 [file a-77-00196-sup2.gif]
