# Supplementary material for: A new electron diffraction approach for structure refinement applied to Ca3Mn2O7
Source: Acta Crystallogr A Found Adv. 2021 Mar 17;77(Pt 3):196–207. doi: 10.1107/S2053273321001546 (PMC8127389; doi:10.1107/S2053273321001546)
Supplement: Supplementary file 3 [file a-77-00196-sup3.pdf]

FOUNDATIONS
ADVANCES

Volume 77 (2021)

Supporting information for article:

A new electron diffraction approach for structure refinement applied to Ca3Mn2O7

R. Beanland, K. Smith, P. Vaněk, H. Zhang, A. Hubert, K. Evans, R.A. Römer and S. Kamba

Supplemental Material for Structural refinement of $Ca_3Mn_2O_7$ using electron diffraction

R.Beanland[a], K.Smith[b], P.Vaněk[c], H.Zhang[d], A.Hubert[a], R.A.Römer[a], S.Kamba[c]

[a]Department of Physics, University of Warwick, Coventry CV4 7AL, UK, [b]DSTL, Oxford, UK, [c]Institute of Physics, Academy of Sciences of the Czech Republic, Na Slovance 2, 182 21 Prague 8, Czech Republic, [d] National Institute of Standards and Technology, Material Measurement Laboratory, 100 Bureau Drive, Gaithersburg, MD 20899 USA

A D-LACBED experiment generates a large amount of data, and matching this data to simulation produces yet more. Here we show results, images and plots that are too numerous to be included in the main body of the article.

S1.1. Refinements at elevated temperature

Site		Coordinate			$B(Å^2)$
Ca1	4a	0.7425(6)	0.2675(7)	0	0.91(2)
Ca2	8b	0.7522(4)	0.2679(5)	0.1863(1)	0.79(1)
Mn	8b	0.2480(5)	0.2500(20)	0.0979(1)	0.52(2)
O1	4a	0.2524(10)	0.2954(11)	0	2.3(1)
O2	8b	0.0240(11)	0.5292(11)	0.1026(2)	1.5(1)
O3	8b	0.9659(11)	0.0280(10)	0.0912(1)	1.5(1)
O4	8b	0.2440(7)	0.2043(6)	0.1967(1)	1.8(1)

Table S1. *Refined 100° C $A2_1am$ structural parameters. Final fit index 3.68% at a specimen thickness of 53 nm.*

Site		Coordinate			$B(Å^2)$
Ca1	4a	0.75	0.2835(4)	0	0.96(2)
Ca2	8b	0.75	0.2759(3)	0.1863(1)	1.04(2)
Mn	8b	0.25	0.2620(10)	0.0976(1)	0.75(2)
O1	4a	0.25	0.2159(11)	0	1.3(1)
O2	8b	0.0318(5)	0.5318(5)	0.0960(1)	2.5(1)
O3	8b	0.9682(5)	0.0318(5)	0.0960(1)	2.5(1)
O4	8b	0.25	0.2043(6)	0.1960(1)	1.7(1)

Table S2. *Refined 200° C $A2_1am$ structural parameters. Final fit index 3.55% at a specimen thickness of 55 nm.*

Site		Coordinate			$B(\text{Å}^2)$
Ca1	4a	0.75	0.2775(8)	0	1.2(5)
Ca2	8b	0.75	0.2711(5)	0.1861(1)	1.06(5)
Mn	8b	0.25	0.2550(20)	0.0976(1)	0.76(4)
O1	4a	0.25	0.2939(9)	0	0.8(1)
O2	8b	0.0267(5)	0.5267(5)	0.0964(1)	2.6(1)
O3	8b	0.9733(5)	0.0267(5)	0.0964(1)	2.6(1)
O4	8b	0.25	0.2164(9)	0.1966(1)	1.0(1)

Table S3. *Refined 300° C $A2_1am$ structural parameters. Final fit index 2.79% at a specimen thickness of 53 nm.*

Site		Coordinate			$B(\text{Å}^2)$
Ca1	4a	0.75	0.2735(8)	0	1.65(5)
Ca2	8b	0.75	0.2692(5)	0.1865(1)	1.59(5)
Mn	8b	0.25	0.2500(20)	0.0978(1)	0.88(4)
O1	4a	0.25	0.2870(11)	0	2.3(1)
O2	8b	0.0289(5)	0.5289(5)	0.0962(1)	2.2(1)
O3	8b	0.9711(5)	0.0289(5)	0.0962(1)	2.4(1)
O4	8b	0.25	0.2129(9)	0.1965(1)	2.2(1)

Table S4. *Refined 400° C $A2_1am$ structural parameters. Final fit index 3.62% at a specimen thickness of 59 nm.*

S1.2. Error estimates

The displacement of any atom from the best-fit refinement produces an increase in fit index. For small displacements the fit index increases parabolically as shown in Fig. S1. Ten refinements of atomic coordinates in RT data were performed with starting points ±0.001 from the initial best fit refinement. The standard deviation of these refinements are shown in Fig. S1 plotted against the error estimated from a change in fit parameter of 10^{-5}. The equivalence of the two measures allows the error in a refined parameter to be estimated simply from the sensitivity.

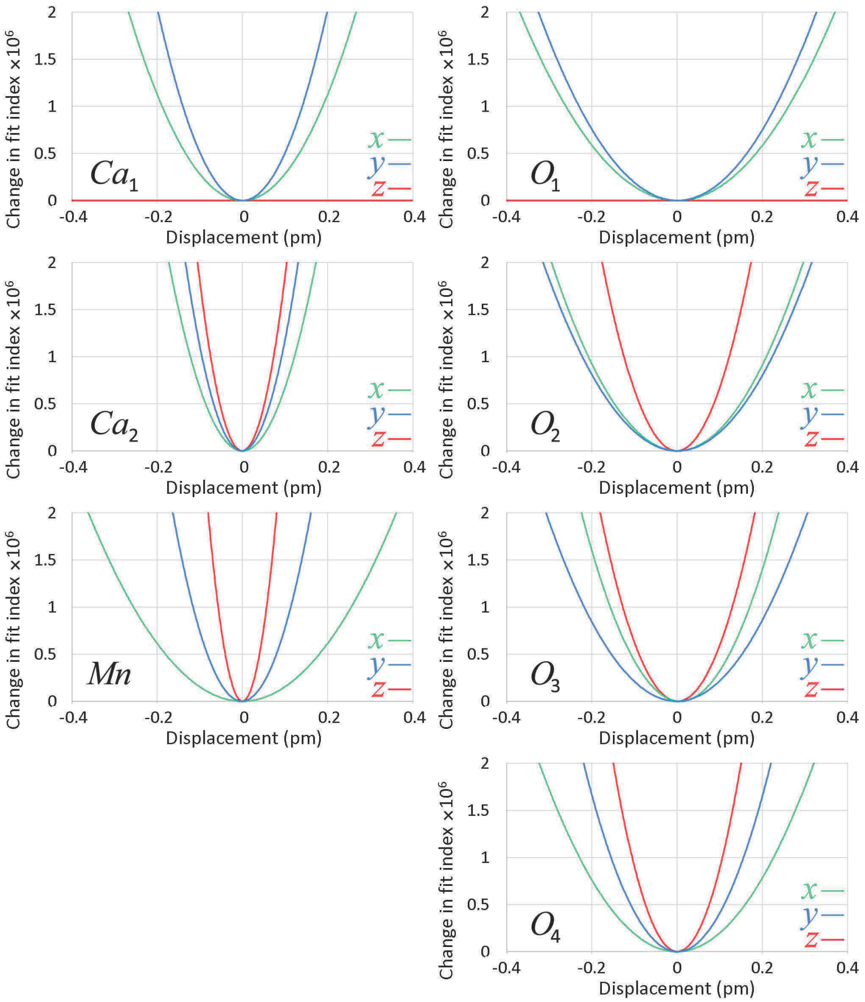

Fig. S1. Parabolic fits to the change in fit parameter produced by atomic displacements.

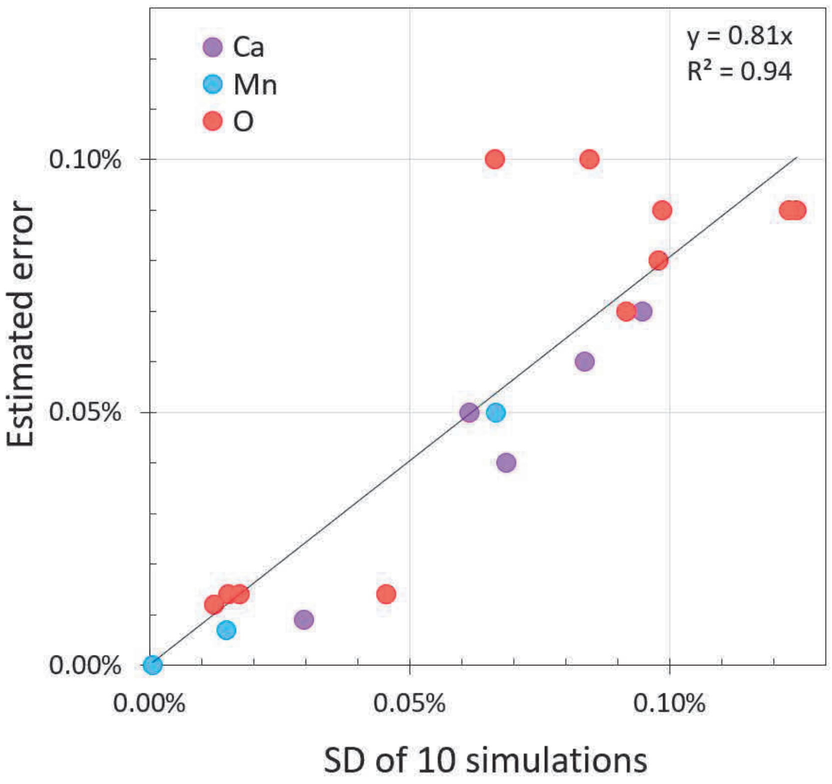

Fig. S2. Standard deviation of atomic coordinates from multiple refinements compared with the change in atomic coordinate produced by a change in fit parameter of 10^{-5}.

S1.3. Deltas from structural refinement

Normalised difference images (δ_j) and their RMS values Δ_j can be used to give a quantification of the goodness of fit in D-LACBED refinement, similar to the residuals used in Rietveld refinement. Fig. S3 shows the difference between the starting point for the RT refinement and experimental data. The values decrease for all patterns j to values of $\sim$ 0.15 or below, apart from 111, $11\bar{1}1$ and $15\bar{1}1$.

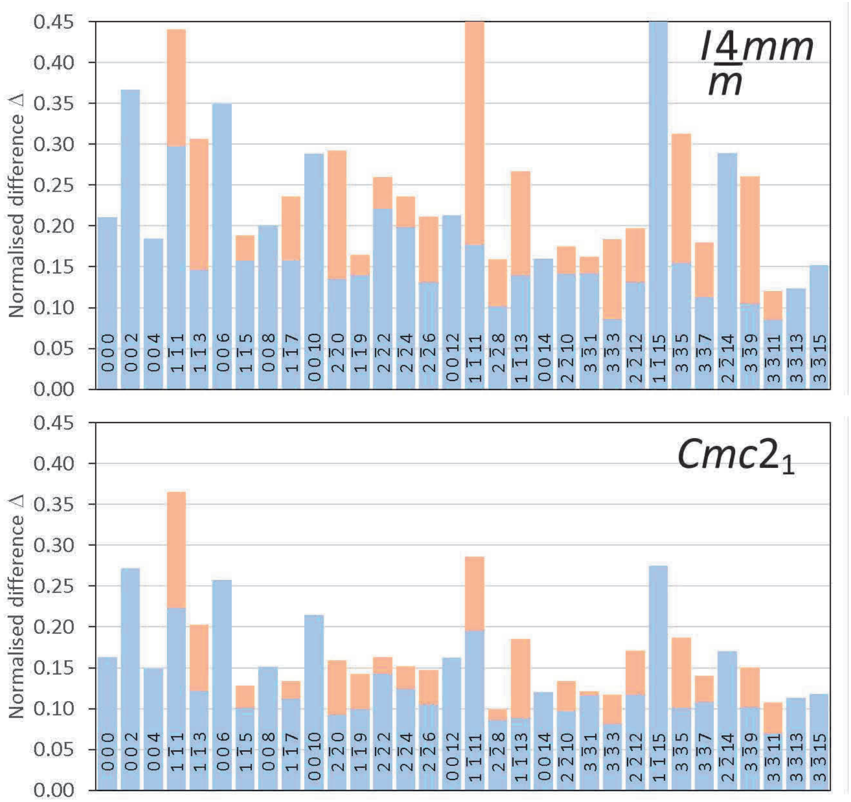

Fig. S3. Δ_j values for the difference between simulation and experiment for all reflections j in the RT measurement. Bijvoet pairs $j = \bar{h}hl$ and $j = h\bar{h}l$ are shown with the same indices but different colours (orange and blue). Top: the initial fit for the $I4/mmm$ structure. Bottom: best fit $Cmc2_1$ structure. Note the general improvement in fit for all patterns and large improvements for the worst-fitting patterns such as $1\ \bar{1}\ 11$ and $1\ \bar{1}\ 15$

S1.4. Delta images for different structural parameters

The symmetry of the normalised difference (δ) images can be captured with only three patterns, one from the 001 systematic row and a Bijvoet pair, shown in Fig. S4 for the 19 structural parameters corresponding to coordinate refinement calculated for the $I4/mmm$ structure. Figures S5 to S23 show the full set of δ images for the same 19 structural parameters, calculated at the refined RT $A2_1am$ $Ca_3Mn_2O_7$ structure. (N.B. Because this structure is distorted in comparison with the $I4/mmm$ prototype, the symmetry of these patterns is lower than that shown in Fig. S4).

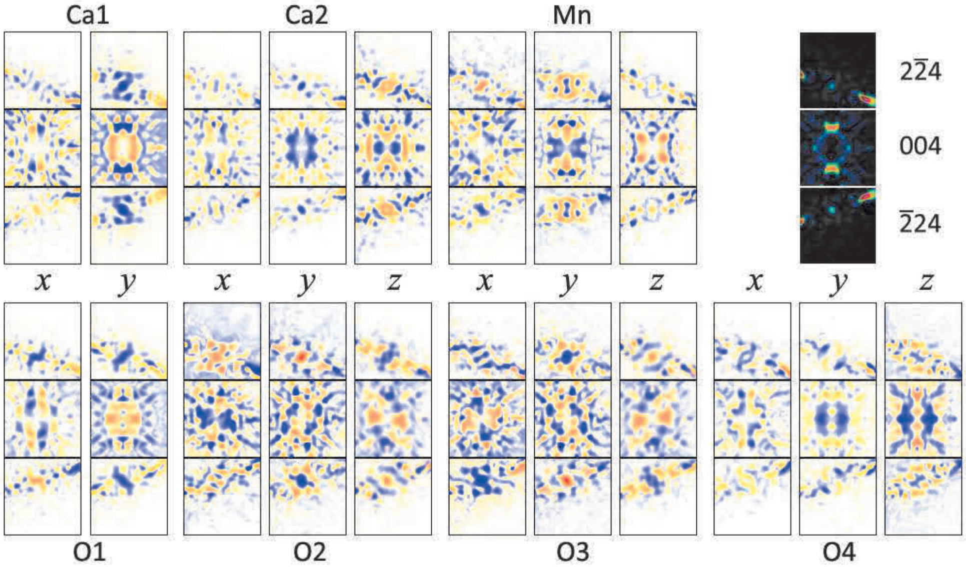

Fig. S4. Changes in the intensity of three D-LACBED patterns (the 004 pattern and the Bijvoet pair $\bar{2}24$, $2\bar{2}4$) for all possible atom displacements in $A2_1am$, i.e. small movements from the prototype $I4/mmm$ structure along $+x$, $+y$, or $+z$. There are 19 unconstrained atom coordinates in the $A2_1am$ $Ca_3Mn_2O_7$ structure. The changes in intensity can be categorised into three types: a) those which maintain (110) mirror symmetry (e.g. Ca1y, Ca2z); b) those which have antimirror $\bar{m}$ symmetry (e.g. Ca1x, Ca2x); and c) those which break mirror symmetry completely (e.g. O2x, O2z). For the latter, movements of different atoms can produce identical (e.g. O2z, O3z) or opposite (e.g. O2x, O3x) changes in intensity.

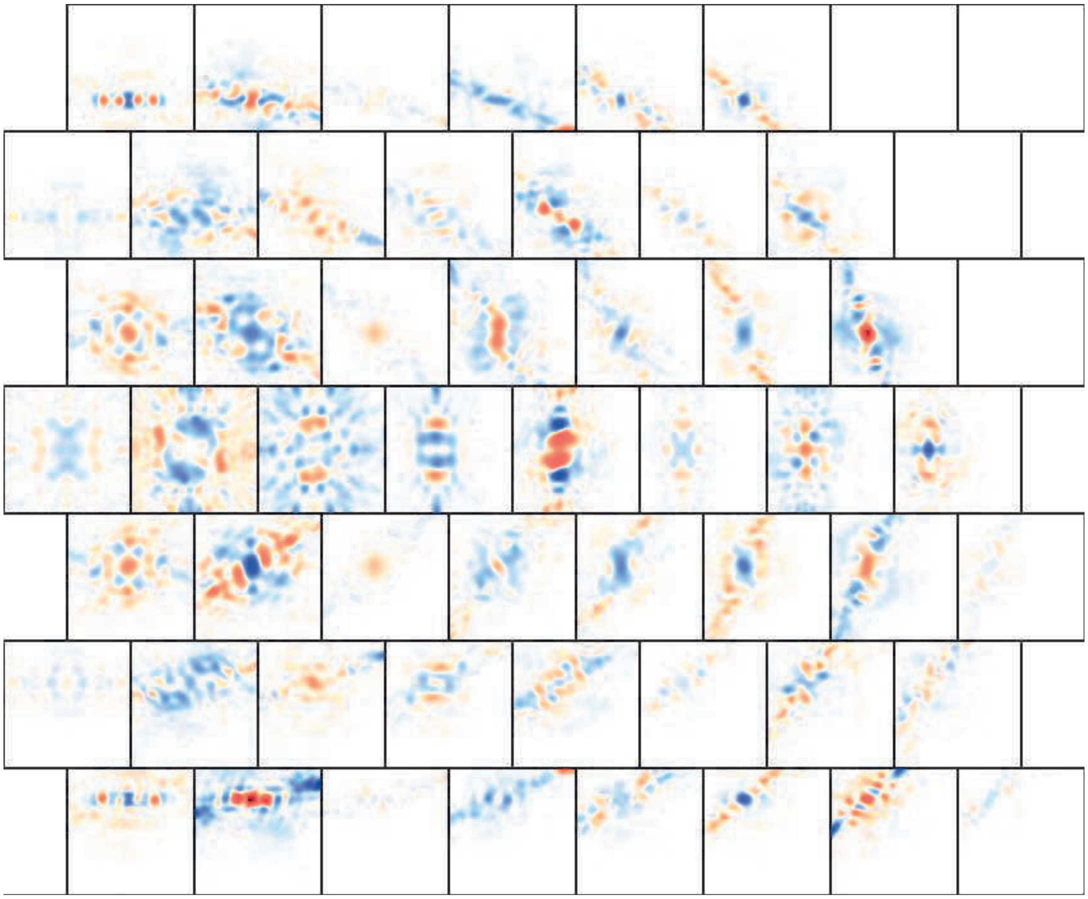

Fig. S5. δ images for the Ca1 y-coordinate at the refined RT $A2_1am$ $Ca_3Mn_2O_7$ structure.

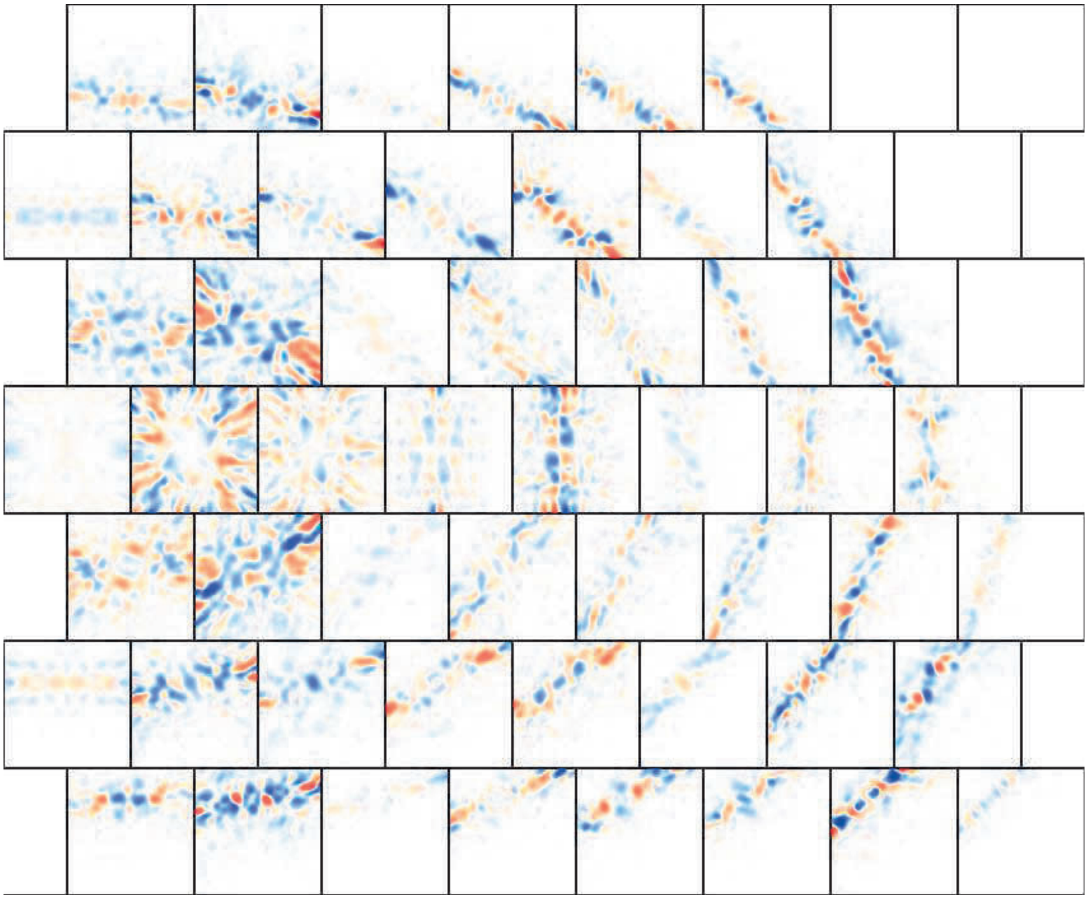

Fig. S6. δ images for the Ca1 x-coordinate at the refined RT $A2_1am$ $Ca_3Mn_2O_7$ structure.

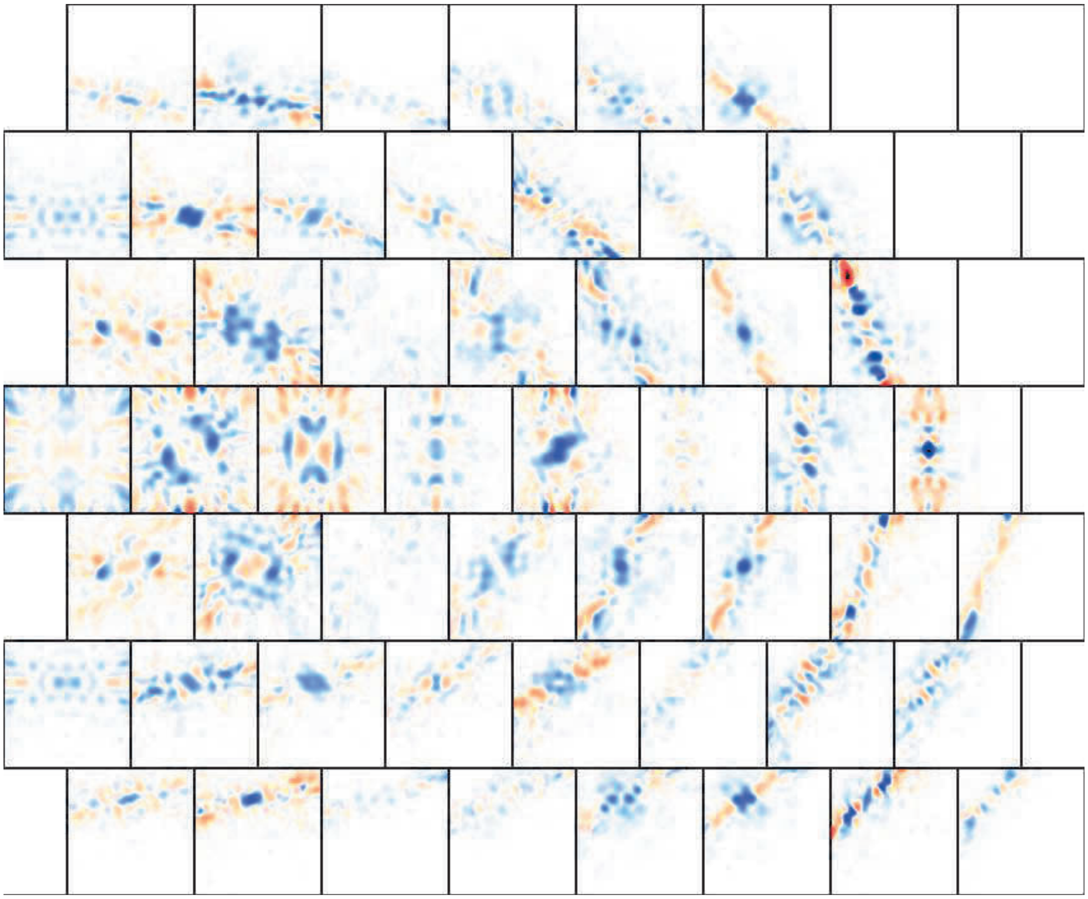

Fig. S7. δ images for the Ca2 z-coordinate at the refined RT $A2_1am$ $Ca_3Mn_2O_7$ structure.

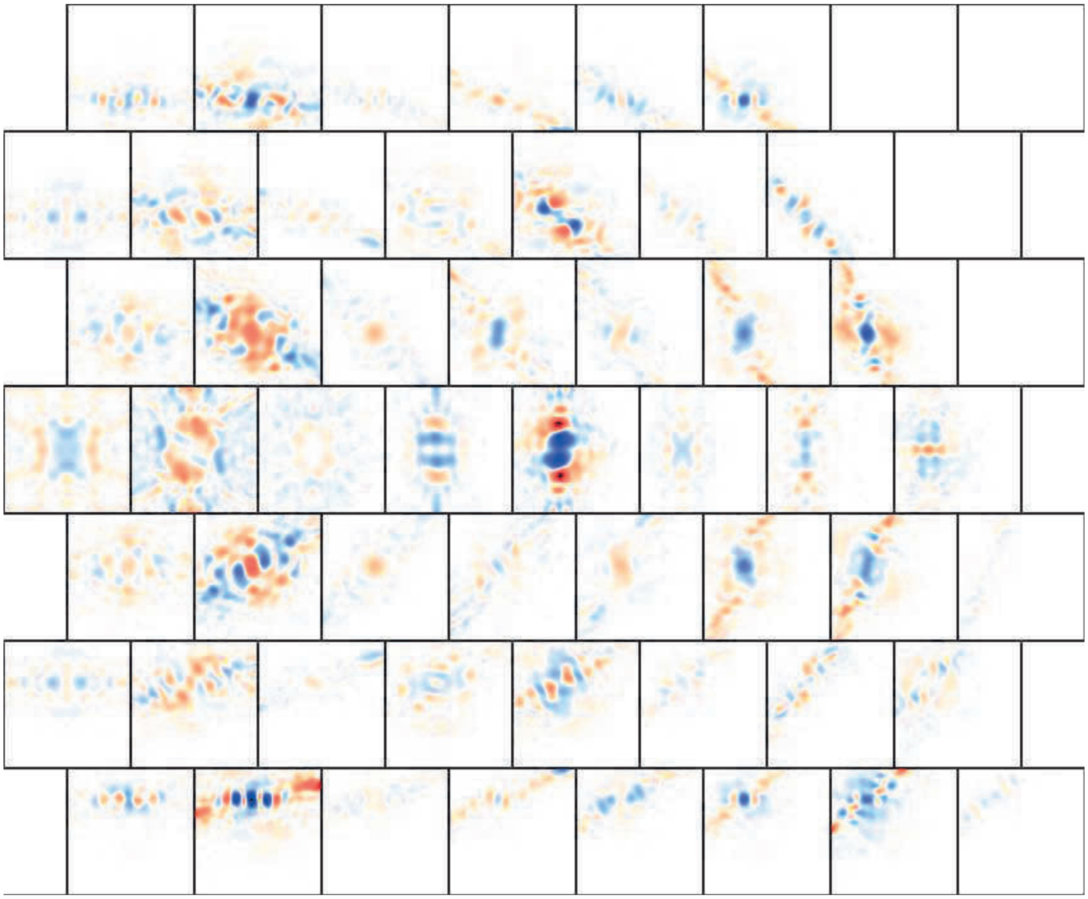

Fig. S8. δ images for the Ca2 y-coordinate at the refined RT $A2_1am$ $Ca_3Mn_2O_7$ structure.

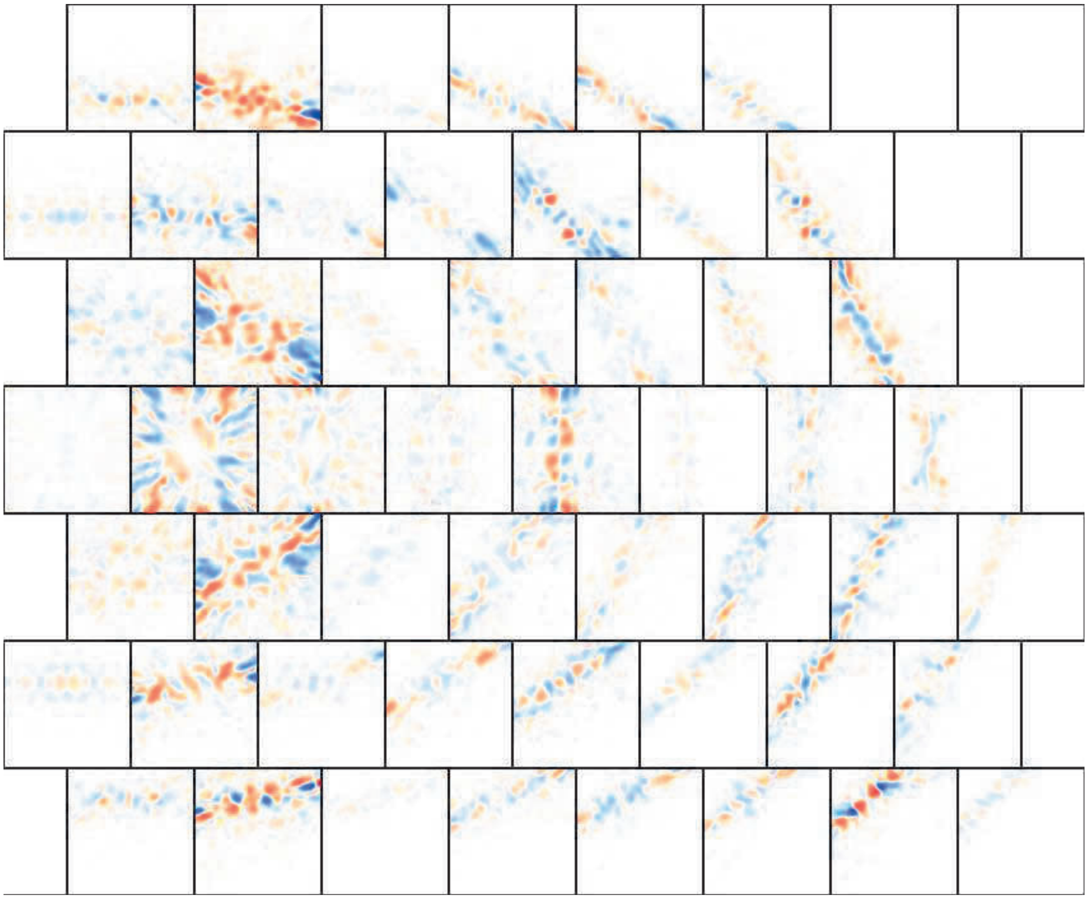

Fig. S9. δ images for the Ca2 x-coordinate at the refined RT $A2_1am$ $Ca_3Mn_2O_7$ structure.

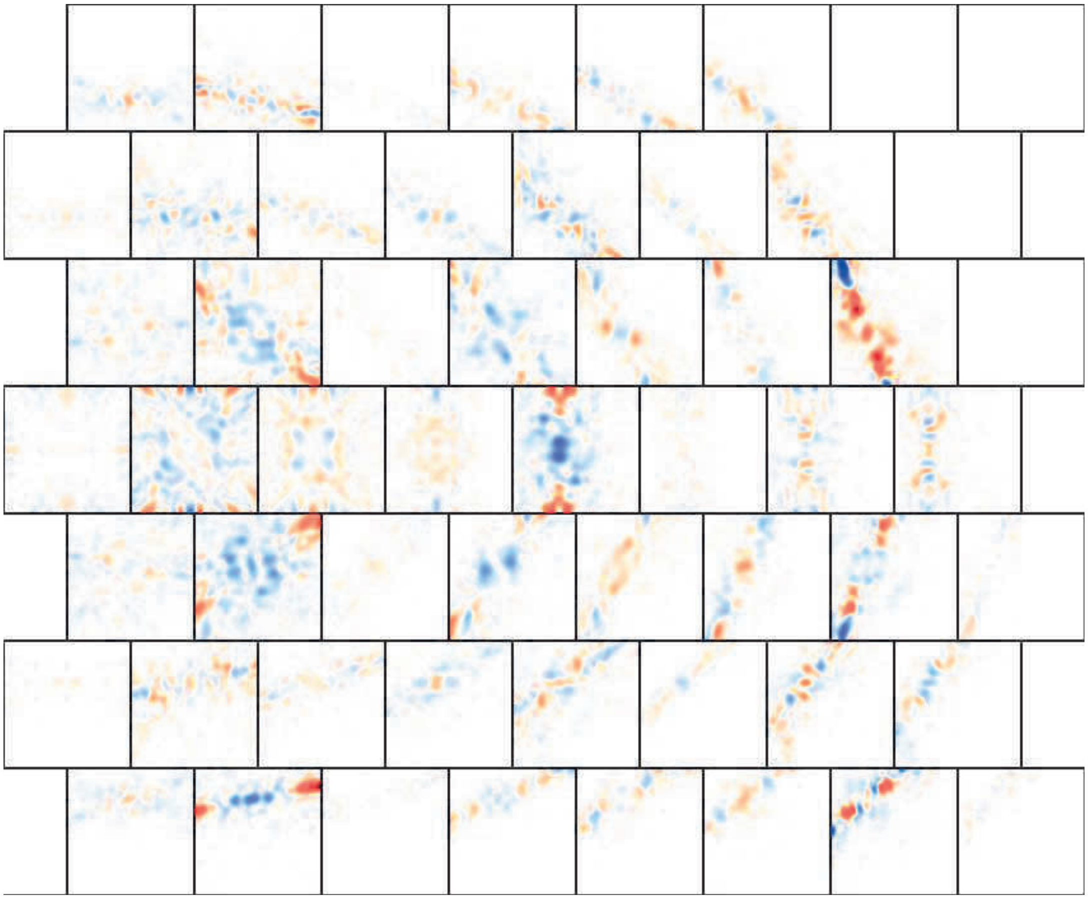

Fig. S10. δ images for the Mn z-coordinate at the refined RT $A2_1am$ $Ca_3Mn_2O_7$ structure.

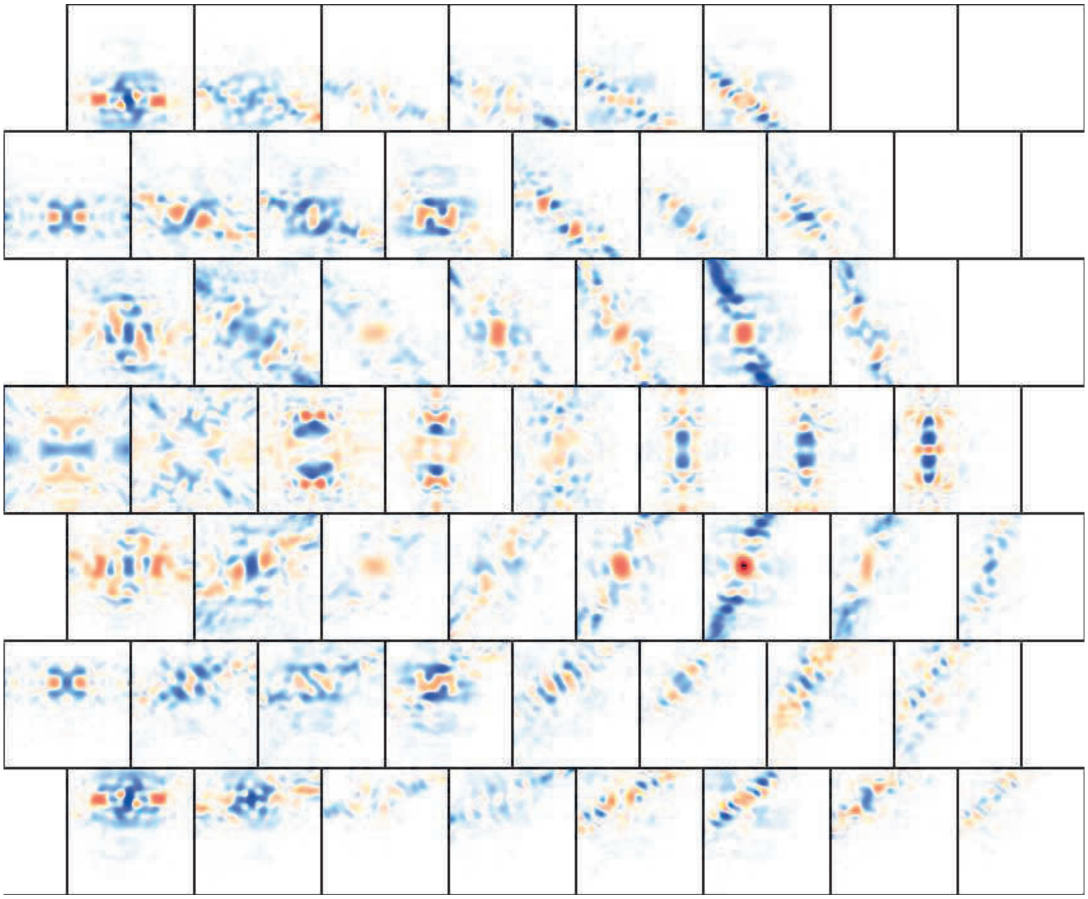

Fig. S11. δ images for the Mn y-coordinate at the refined RT $A2_1am$ $Ca_3Mn_2O_7$ structure.

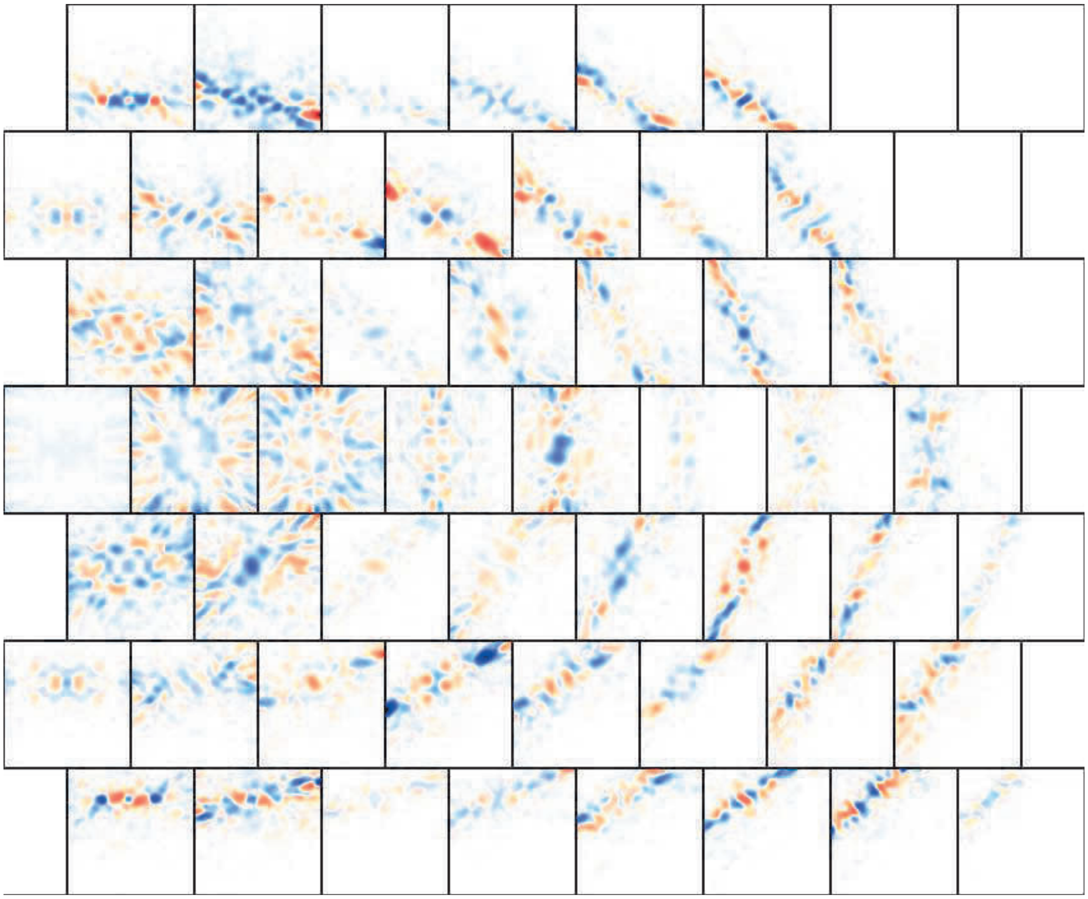

Fig. S12. δ images for the Mn x-coordinate at the refined RT $A2_1am$ $Ca_3Mn_2O_7$ structure.

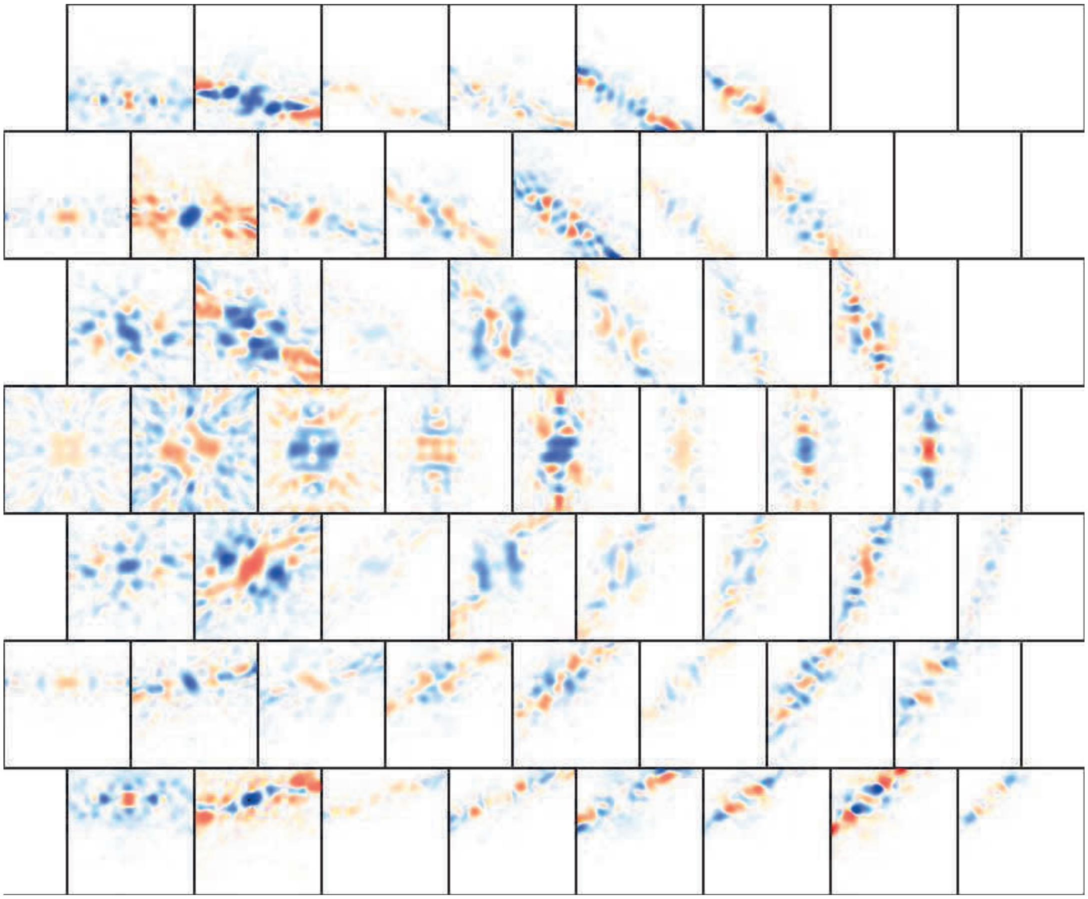

Fig. S13. δ images for the O1 y-coordinate at the refined RT $A2_1am$ $Ca_3Mn_2O_7$ structure.

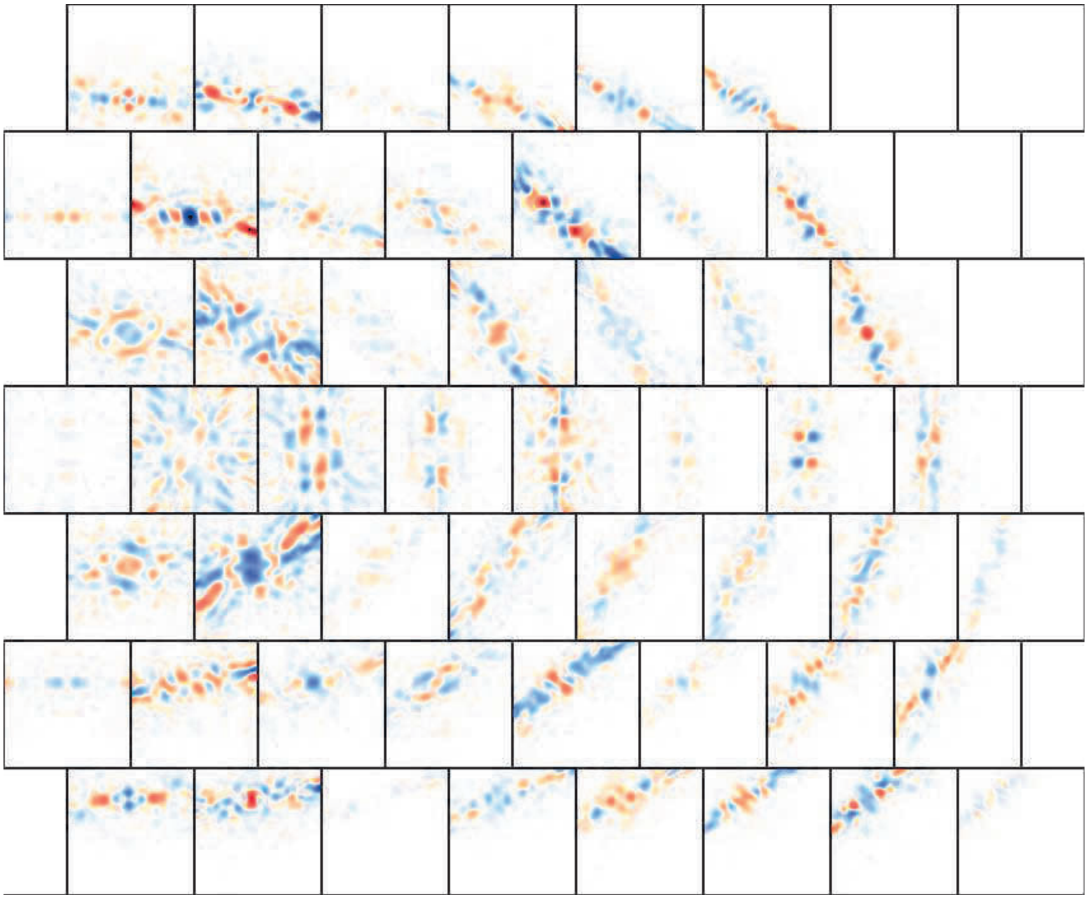

Fig. S14. δ images for the O1 x-coordinate at the refined RT $A2_1am$ $Ca_3Mn_2O_7$ structure.

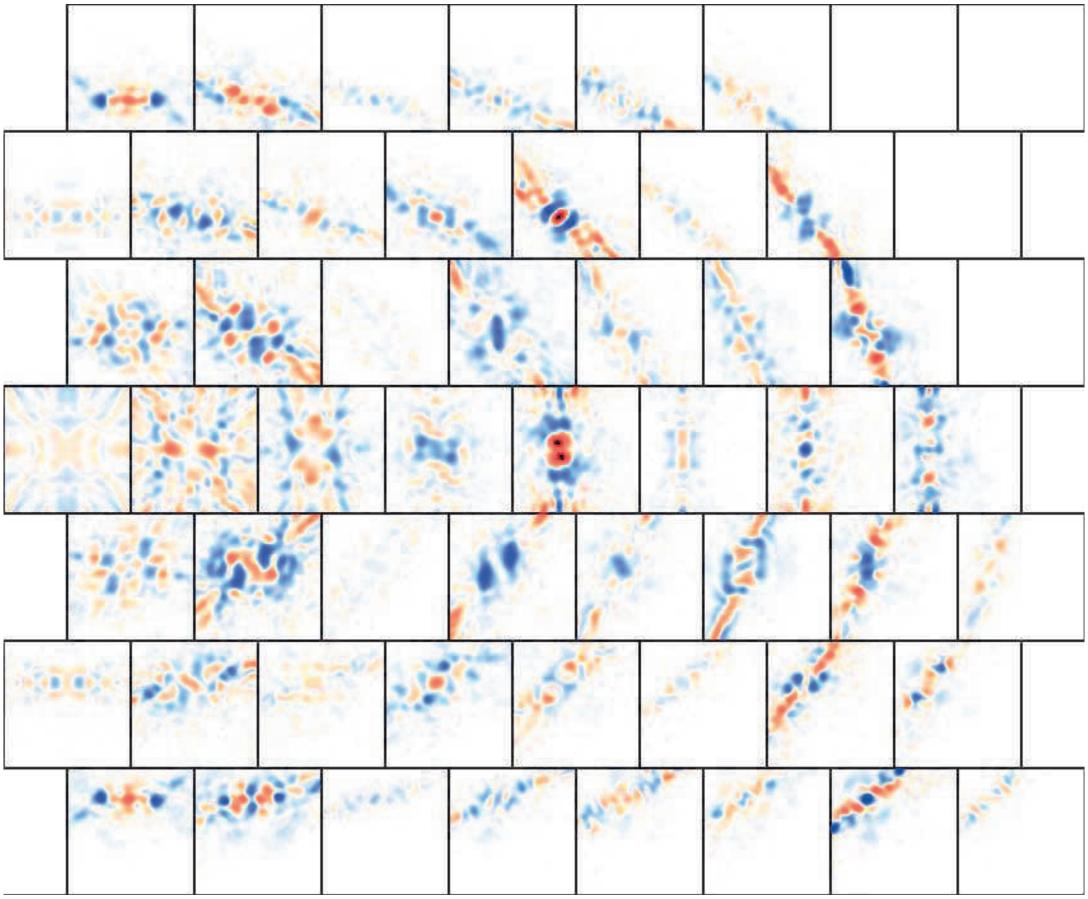

Fig. S15. δ images for the O2 z-coordinate at the refined RT $A2_1am$ $Ca_3Mn_2O_7$ structure.

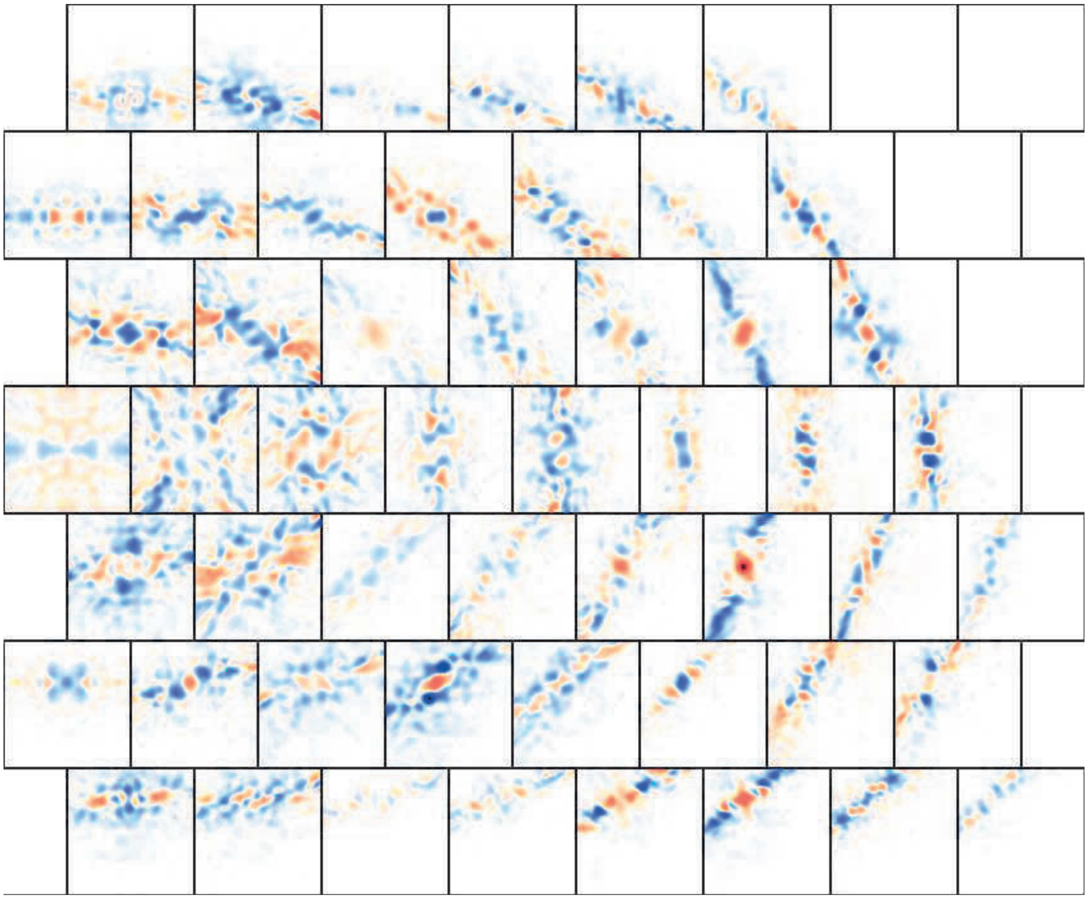

Fig. S16. δ images for the O2 y-coordinate at the refined RT $A2_1am$ $Ca_3Mn_2O_7$ structure.

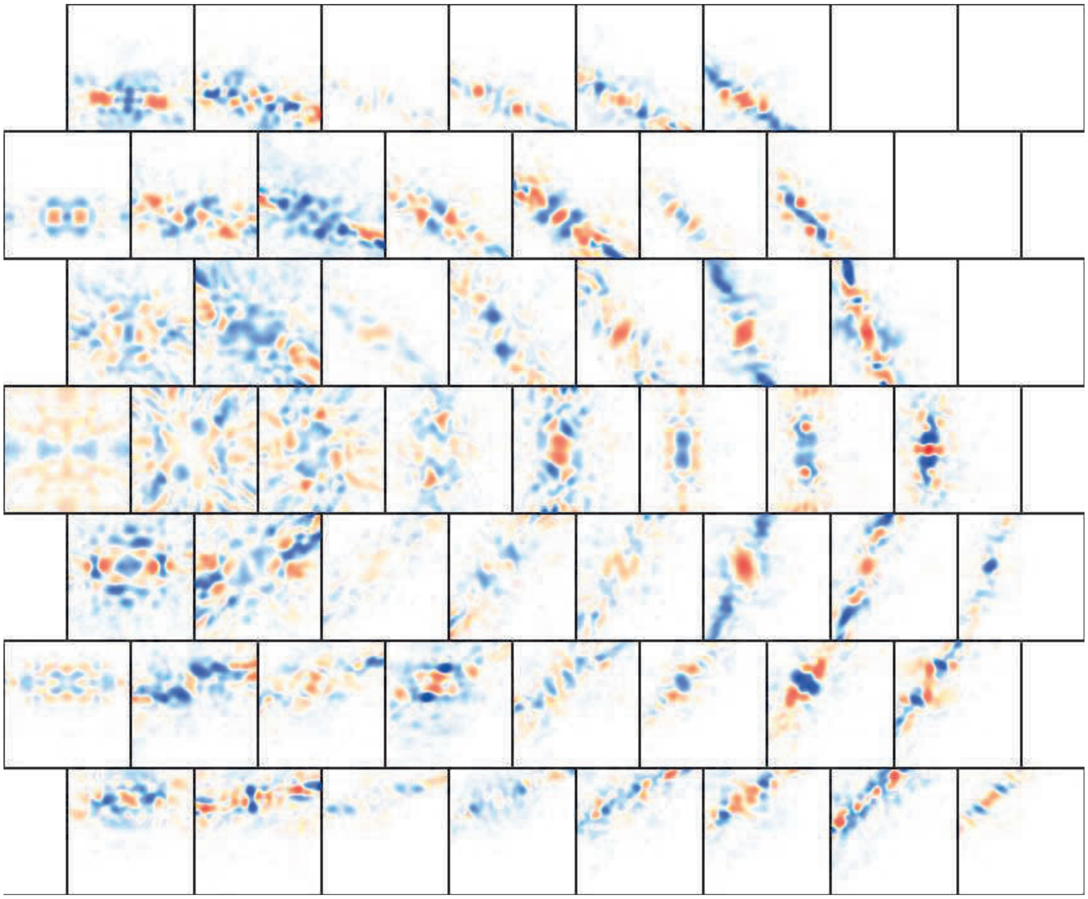

Fig. S17. δ images for the O2 x-coordinate at the refined RT $A2_1am$ $Ca_3Mn_2O_7$ structure.

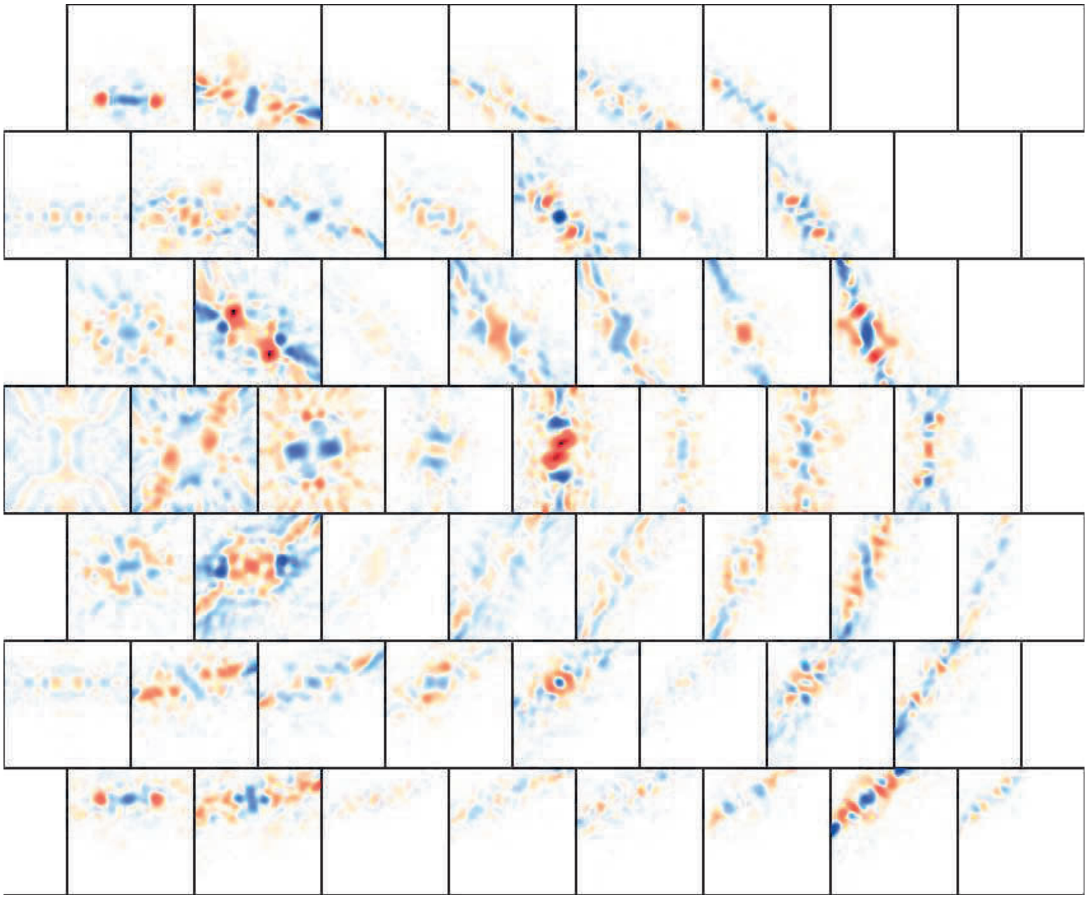

Fig. S18. δ images for the O3 z-coordinate at the refined RT $A2_1am$ $Ca_3Mn_2O_7$ structure.

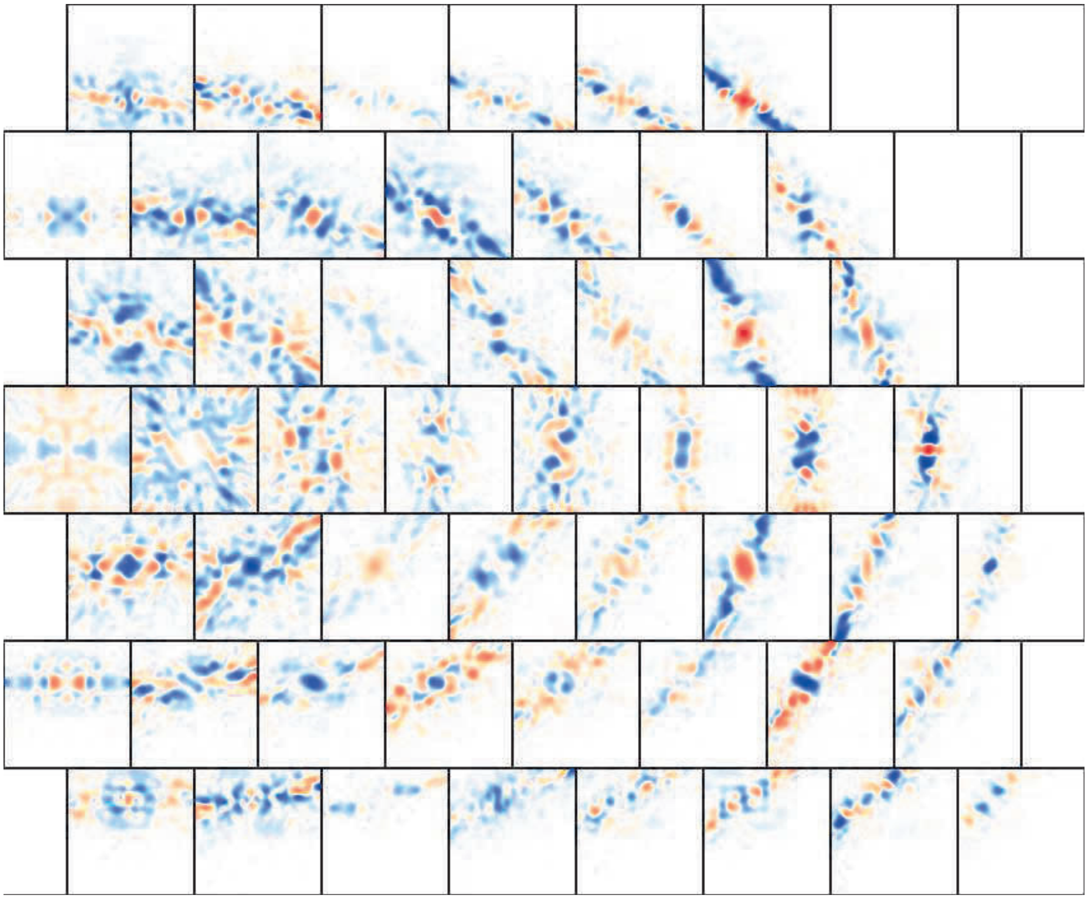

Fig. S19. δ images for the O3 y-coordinate at the refined RT $A2_1am$ $Ca_3Mn_2O_7$ structure.

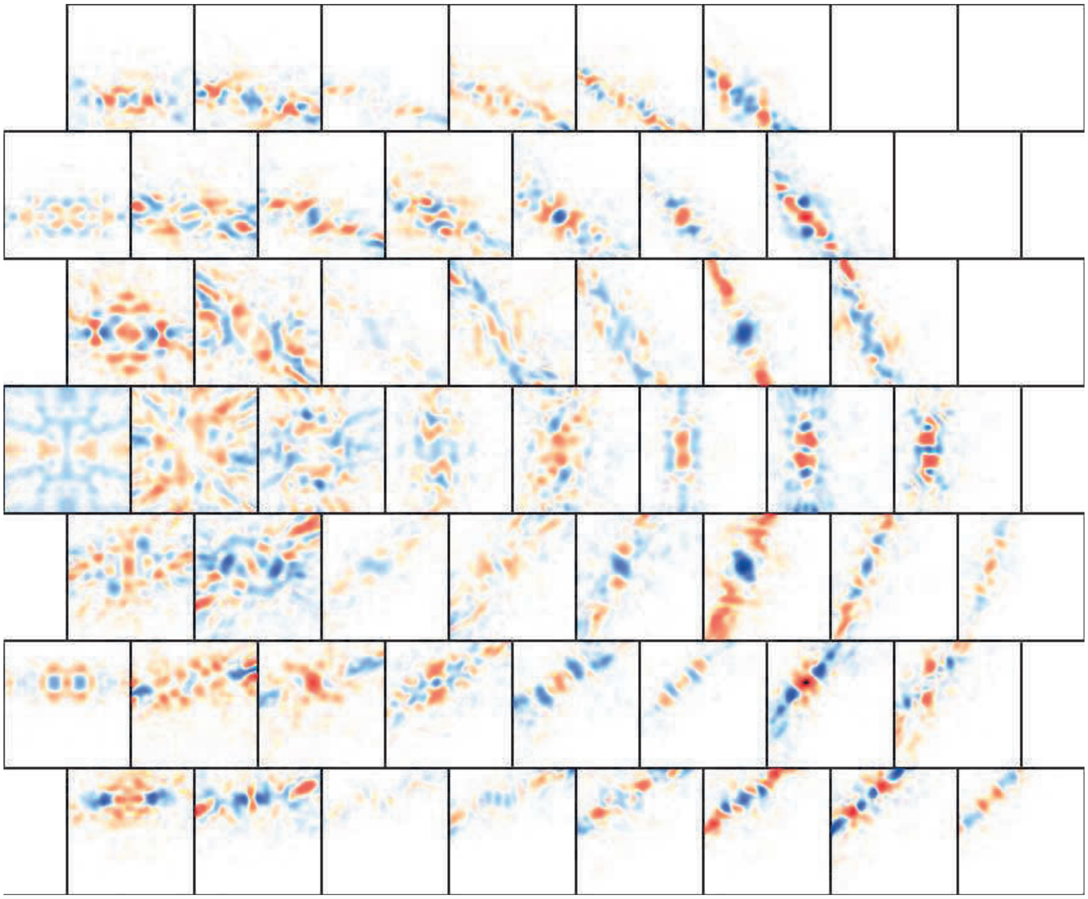

Fig. S20. δ images for the O3 x-coordinate at the refined RT $A2_1am$ $Ca_3Mn_2O_7$ structure.

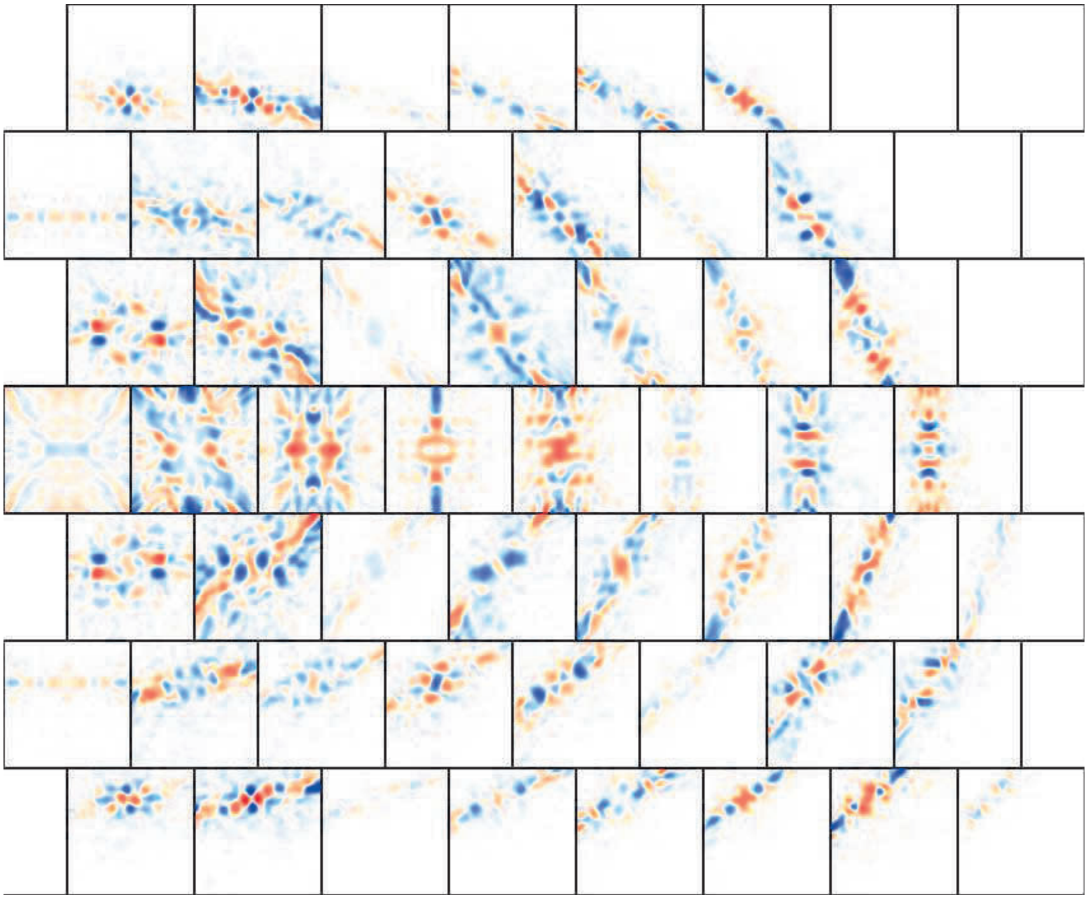

Fig. S21. δ images for the O4 z-coordinate at the refined RT $A2_1am$ $Ca_3Mn_2O_7$ structure.

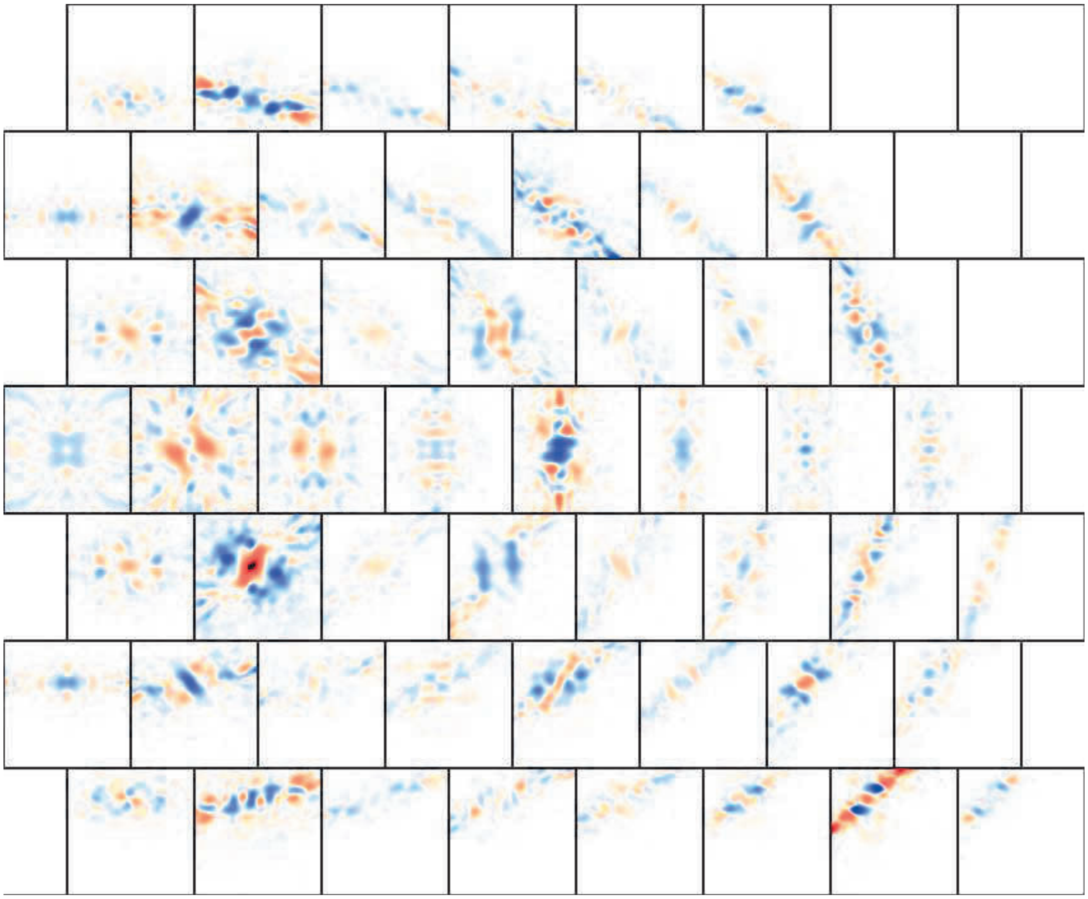

Fig. S22. δ images for the O4 y-coordinate at the refined RT $A2_1am$ $Ca_3Mn_2O_7$ structure.

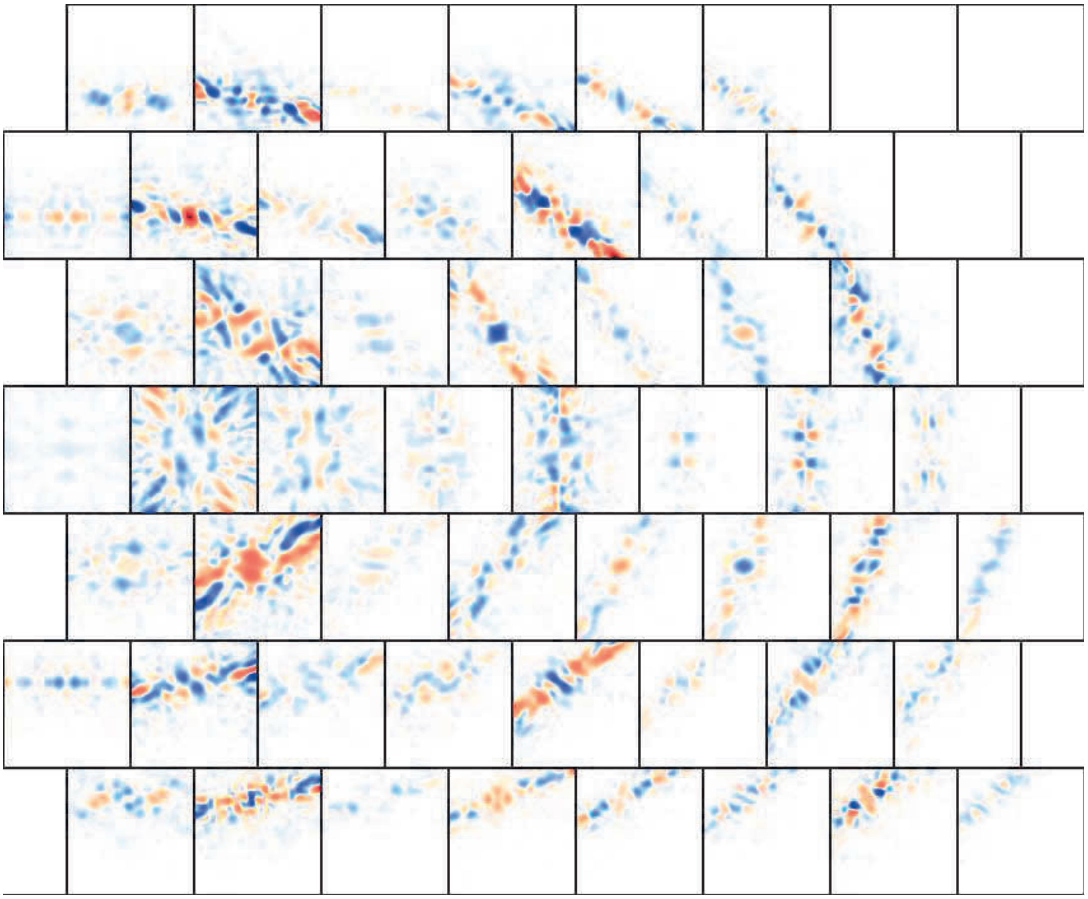

Fig. S23. δ images for the O4 x-coordinate at the refined RT $A2_1am$ $Ca_3Mn_2O_7$ structure.

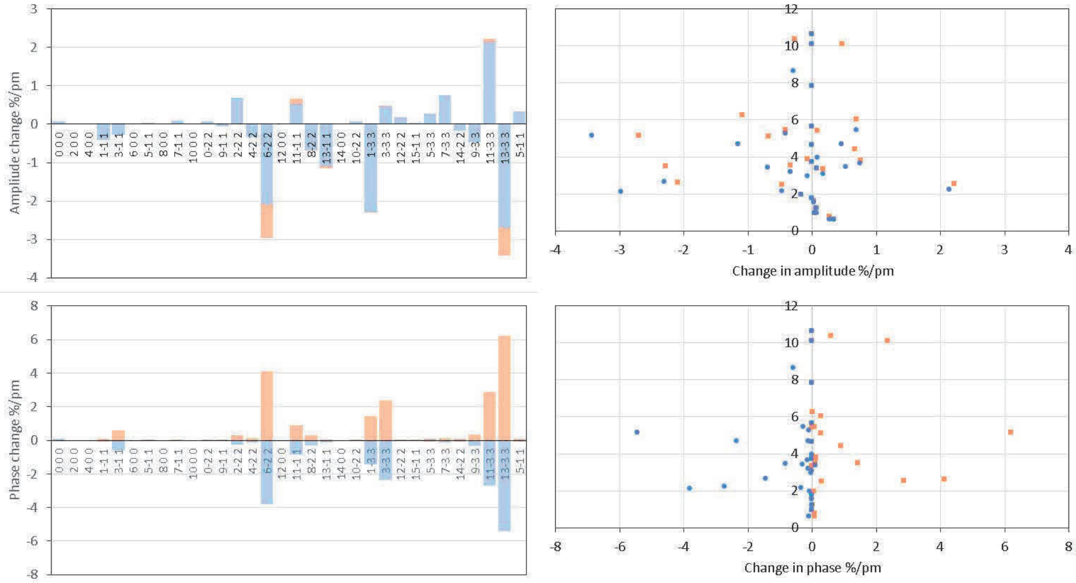

Fig. S24. Left: changes in δF_{ij} amplitude (top) and phase (bottom) of the 54 reflections used in room temperature refinement of the RT $A2_1am$ $Ca_3Mn_2O_7$ structure for a shift of the Ca1 y coordinate of 1 pm. Right: scatter plot relating the change in amplitude and phases from the plots on the left to the change in normalised intensity Δ_{ij}. Bijvoet reflections are colour coded orange and blue.

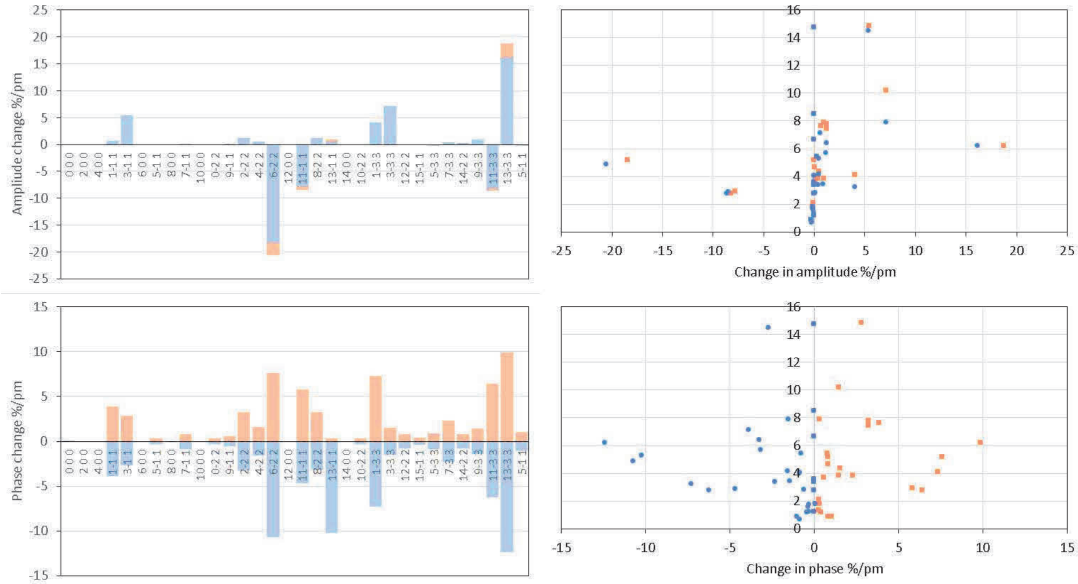

Fig. S25. Left: changes in δF_{ij} amplitude (top) and phase (bottom) of the 54 reflections used in room temperature refinement of the RT $A2_1am$ $Ca_3Mn_2O_7$ structure for a shift of the Ca1 x coordinate of 1 pm. Right: scatter plot relating the change in amplitude and phases from the plots on the left to the change in normalised intensity Δ_{ij}. Bijvoet reflections are colour coded orange and blue.

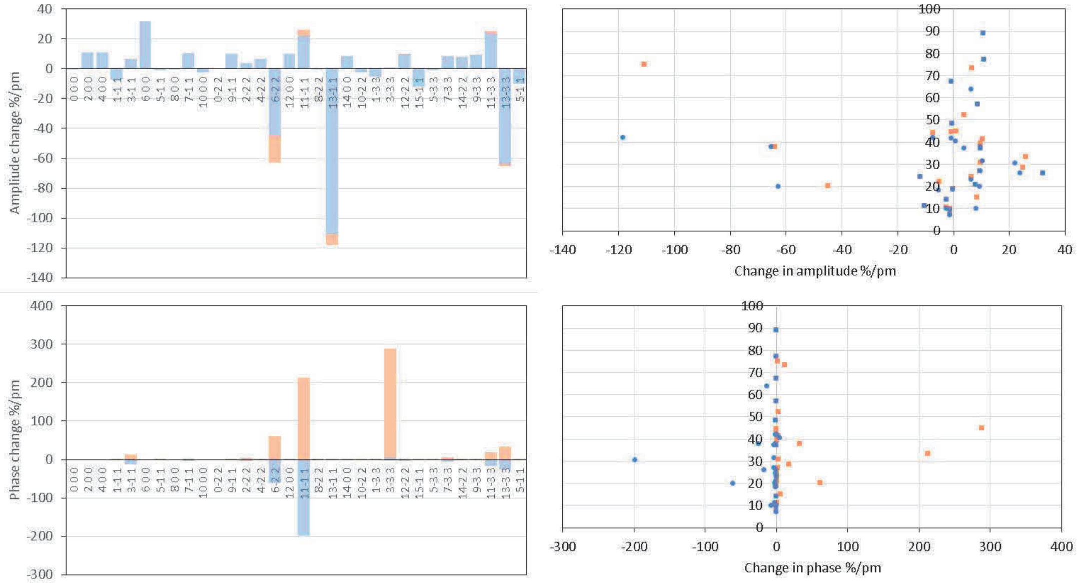

Fig. S26. Left: changes in δF_{ij} amplitude (top) and phase (bottom) of the 54 reflections used in room temperature refinement of the RT $A2_1am$ $Ca_3Mn_2O_7$ structure for a shift of the Ca2 z coordinate of 1 pm. Right: scatter plot relating the change in amplitude and phases from the plots on the left to the change in normalised intensity Δ_{ij}. Bijvoet reflections are colour coded orange and blue.

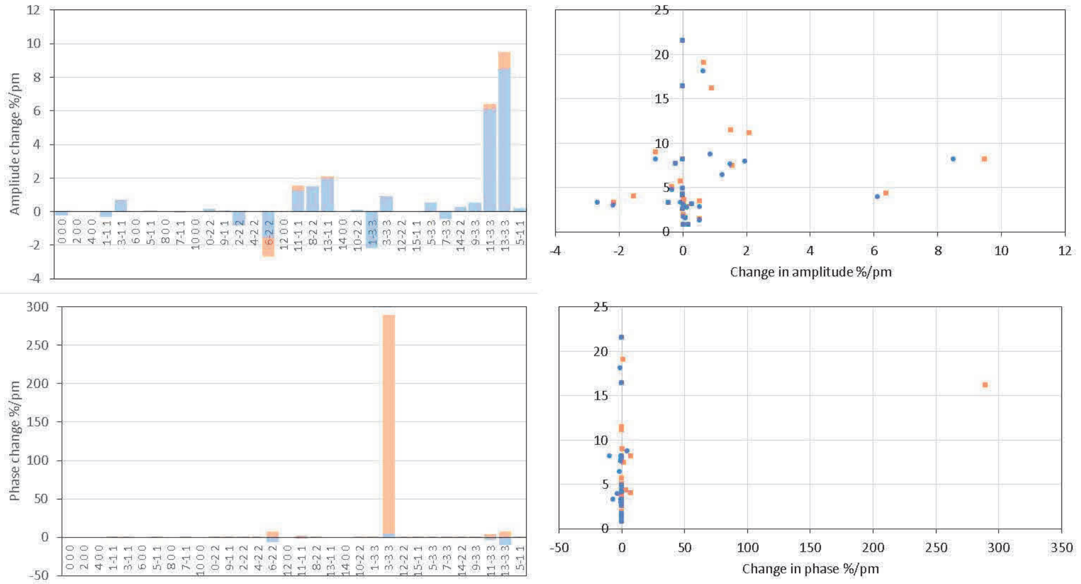

Fig. S27. Left: changes in δF_{ij} amplitude (top) and phase (bottom) of the 54 reflections used in room temperature refinement of the RT $A2_1am$ $Ca_3Mn_2O_7$ structure for a shift of the Ca2 y coordinate of 1 pm. Right: scatter plot relating the change in amplitude and phases from the plots on the left to the change in normalised intensity Δ_{ij}. Bijvoet reflections are colour coded orange and blue.

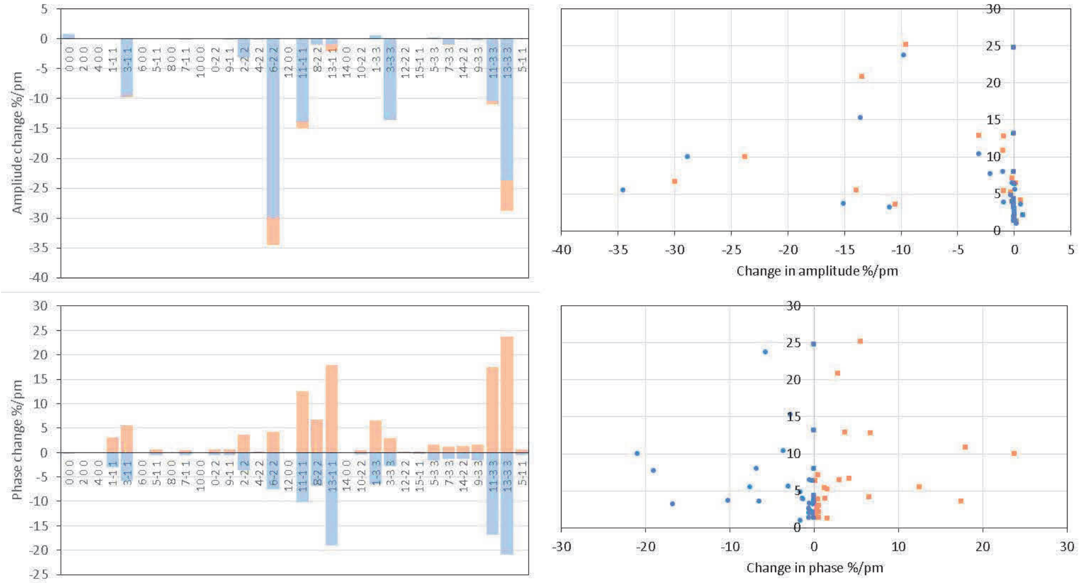

Fig. S28. Left: changes in δF_{ij} amplitude (top) and phase (bottom) of the 54 reflections used in room temperature refinement of the RT $A2_1am$ $Ca_3Mn_2O_7$ structure for a shift of the Ca2 x coordinate of 1 pm. Right: scatter plot relating the change in amplitude and phases from the plots on the left to the change in normalised intensity Δ_{ij}. Bijvoet reflections are colour coded orange and blue.

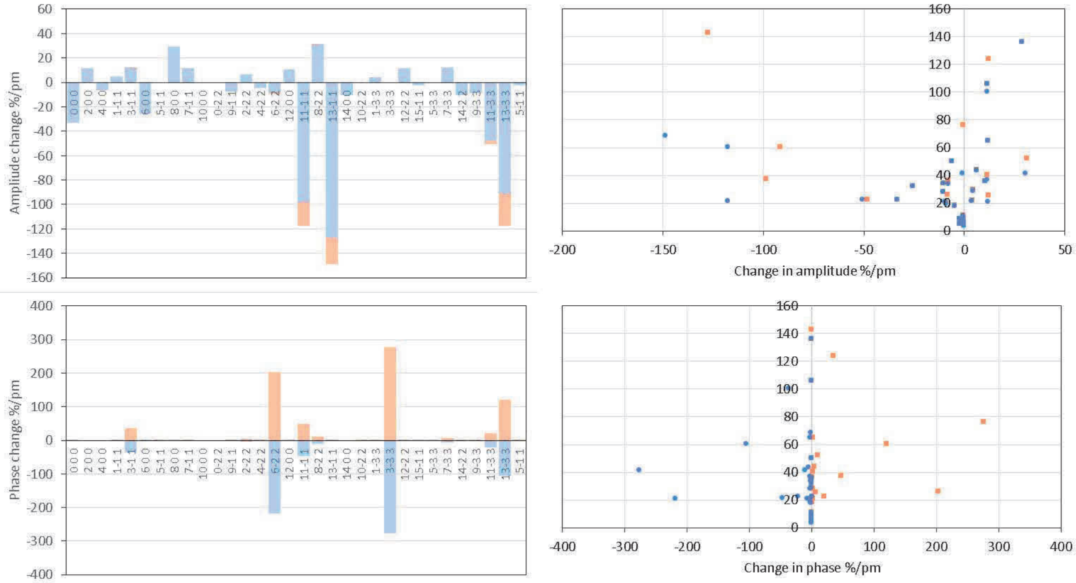

Fig. S29. Left: changes in δF_{ij} amplitude (top) and phase (bottom) of the 54 reflections used in room temperature refinement of the RT $A2_1am$ $Ca_3Mn_2O_7$ structure for a shift of the Mn z coordinate of 1 pm. Right: scatter plot relating the change in amplitude and phases from the plots on the left to the change in normalised intensity Δ_{ij}. Bijvoet reflections are colour coded orange and blue.

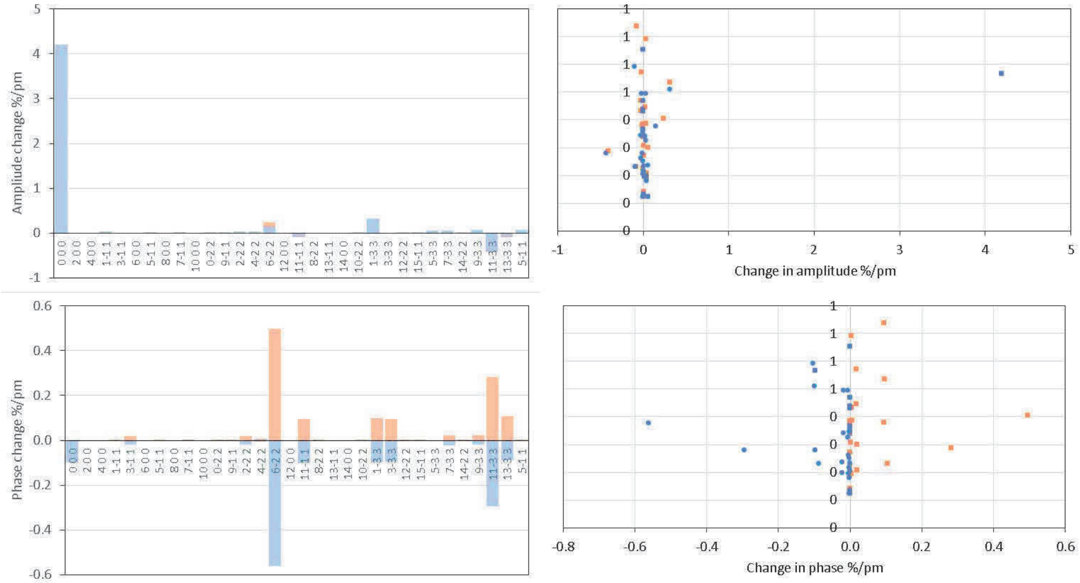

Fig. S30. Left: changes in δF_{ij} amplitude (top) and phase (bottom)of the 54 reflections used in room temperature refinement of the RT $A2_1am$ $Ca_3Mn_2O_7$ structure for a shift of the Mn y coordinate of 1 pm. Right: scatter plot relating the change in amplitude and phases from the plots on the left to the change in normalised intensity Δ_{ij}. Bijvoet reflections are colour coded orange and blue.

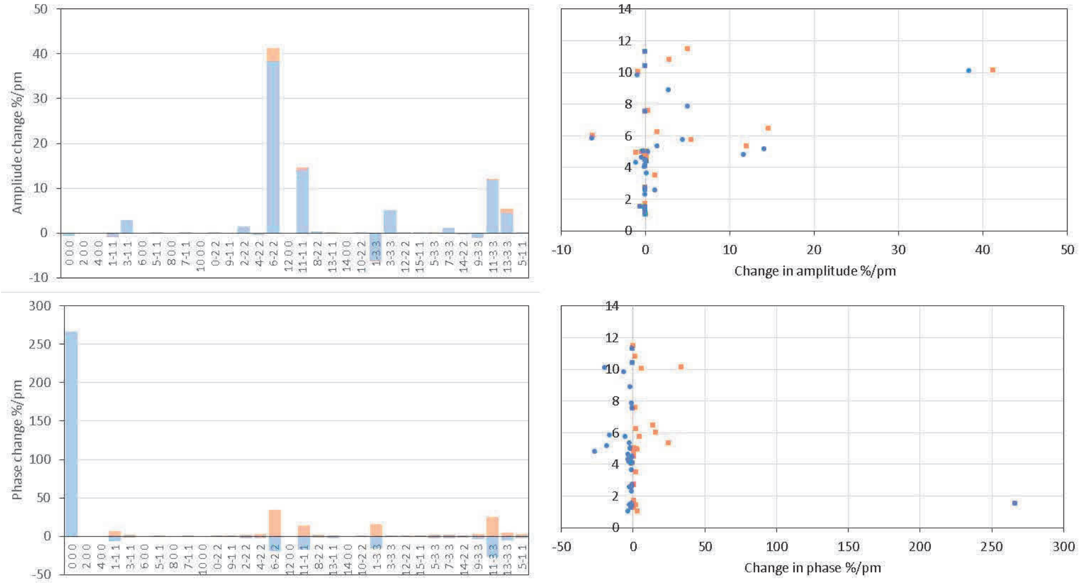

Fig. S31. Left: changes in δF_{ij} amplitude (top) and phase (bottom)of the 54 reflections used in room temperature refinement of the RT $A2_1am$ $Ca_3Mn_2O_7$ structure fo a shift of the Mn x coordinate of 1 pm. Right: scatter plot relating the change in amplitude and phases from the plots on the left to the change in normalised intensity Δ_{ij}. Bijvoet reflections are colour coded orange and blue.

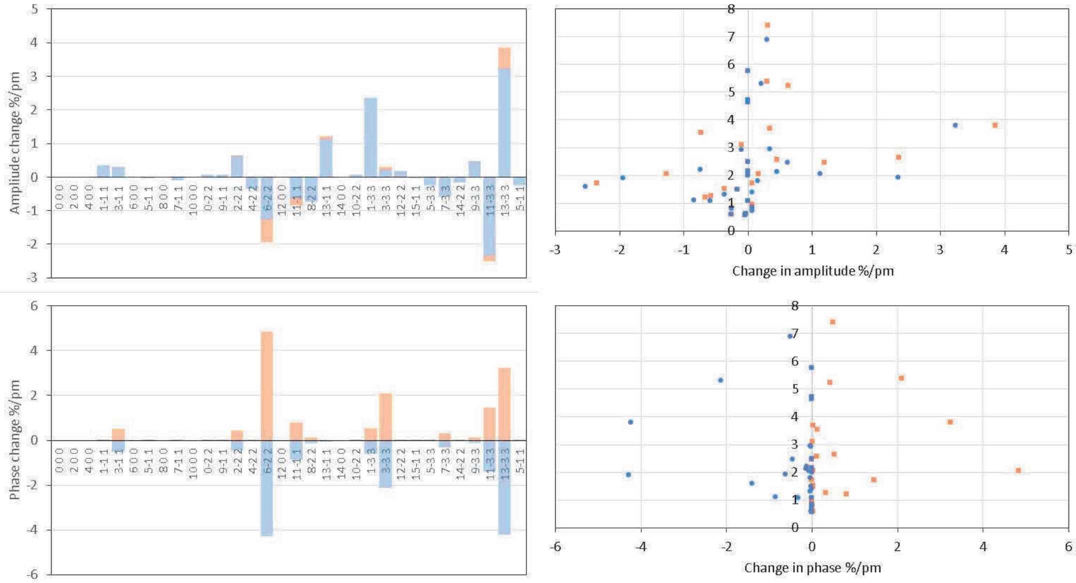

Fig. S32. Left: changes in δF_{ij} amplitude (top) and phase (bottom)of the 54 reflections used in room temperature refinement of the RT $A2_1am$ $Ca_3Mn_2O_7$ structure for a shift of the O1 y coordinate of 1 pm. Right: scatter plot relating the change in amplitude and phases from the plots on the left to the change in normalised intensity Δ_{ij}. Bijvoet reflections are colour coded orange and blue.

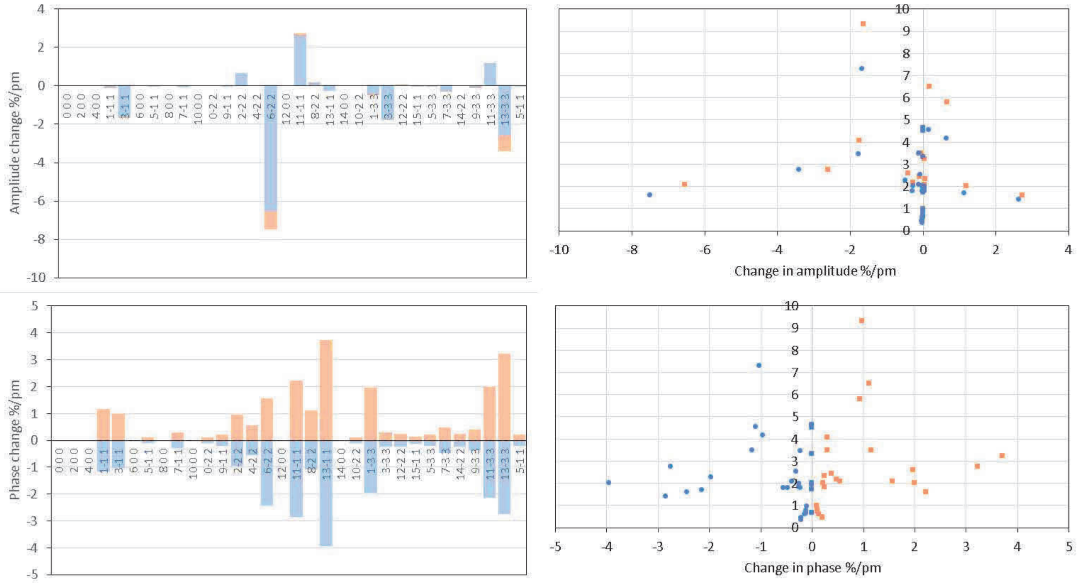

Fig. S33. Left: changes in δF_{ij} amplitude (top) and phase (bottom)of the 54 reflections used in room temperature refinement of the RT $A2_1am$ $Ca_3Mn_2O_7$ structure for a shift of the O1 x coordinate of 1 pm. Right: scatter plot relating the change in amplitude and phases from the plots on the left to the change in normalised intensity Δ_{ij}. Bijvoet reflections are colour coded orange and blue.

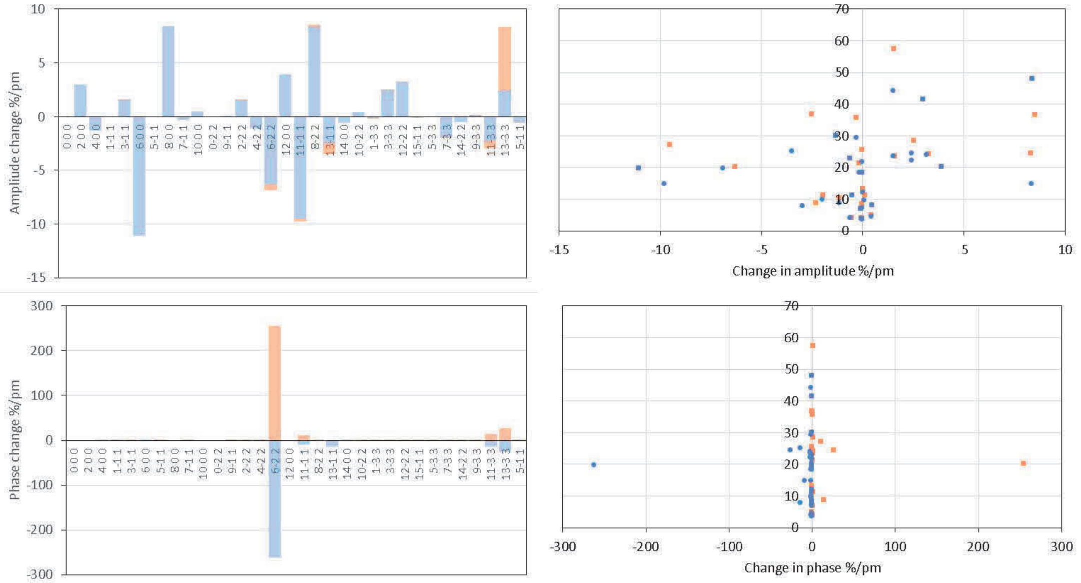

Fig. S34. Left: changes in δF_{ij} amplitude (top) and phase (bottom)of the 54 reflections used in room temperature refinement of the RT $A2_1am$ $Ca_3Mn_2O_7$ structure for a shift of the O2 z coordinate of 1 pm. Right: scatter plot relating the change in amplitude and phases from the plots on the left to the change in normalised intensity Δ_{ij}. Bijvoet reflections are colour coded orange and blue.

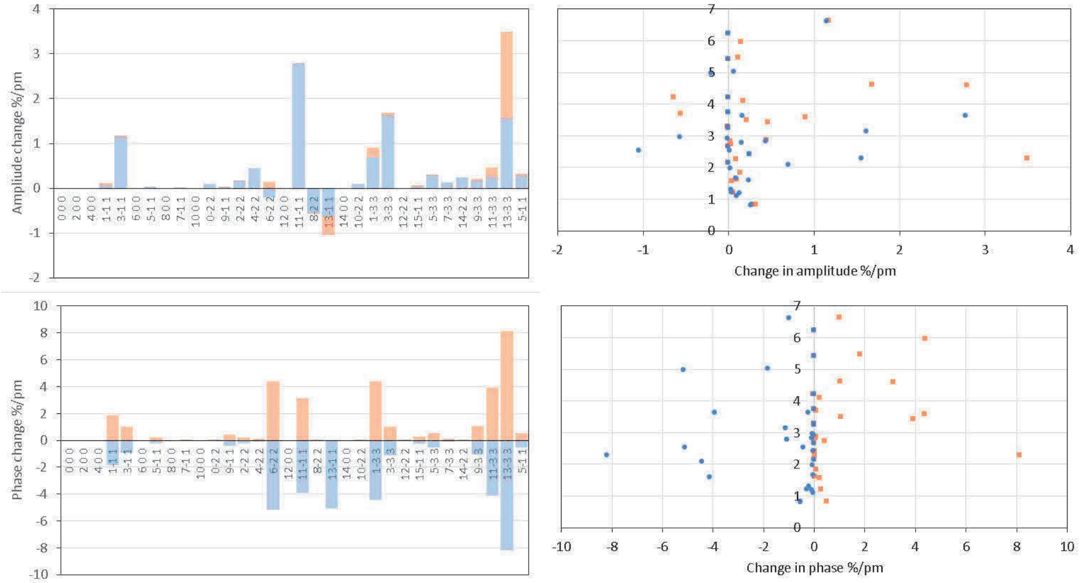

Fig. S35. Left: changes in δF_{ij} amplitude (top) and phase (bottom)of the 54 reflections used in room temperature refinement of the RT $A2_1am$ $Ca_3Mn_2O_7$ structure for a shift of the O2 y coordinate of 1 pm. Right: scatter plot relating the change in amplitude and phases from the plots on the left to the change in normalised intensity Δ_{ij}. Bijvoet reflections are colour coded orange and blue.

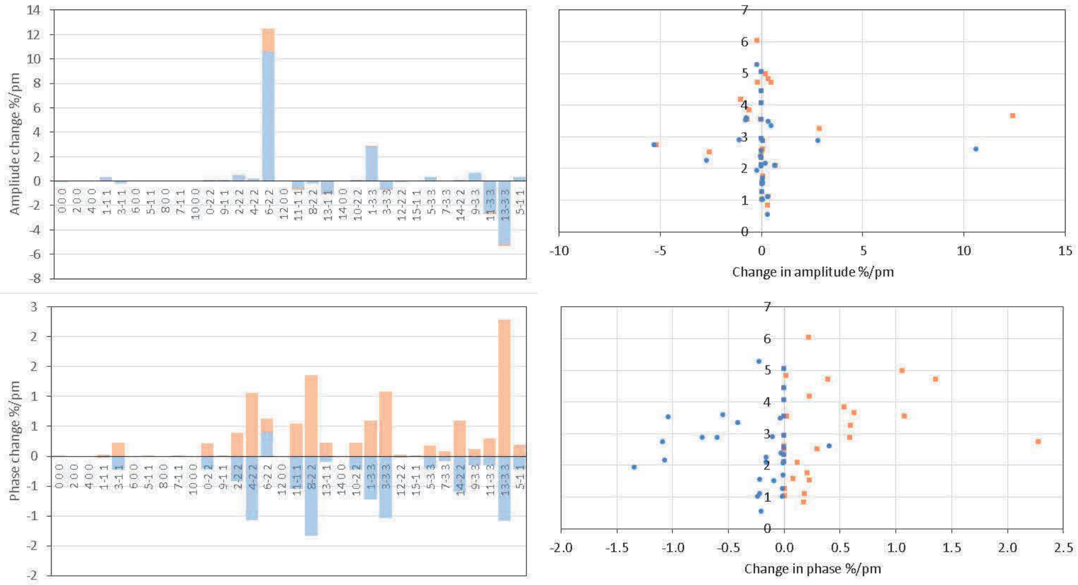

Fig. S36. Left: changes in δF_{ij} amplitude (top) and phase (bottom)of the 54 reflections used in room temperature refinement of the RT $A2_1am$ $Ca_3Mn_2O_7$ structure for a shift of the O2 x coordinate of 1 pm. Right: scatter plot relating the change in amplitude and phases from the plots on the left to the change in normalised intensity Δ_{ij}. Bijvoet reflections are colour coded orange and blue.

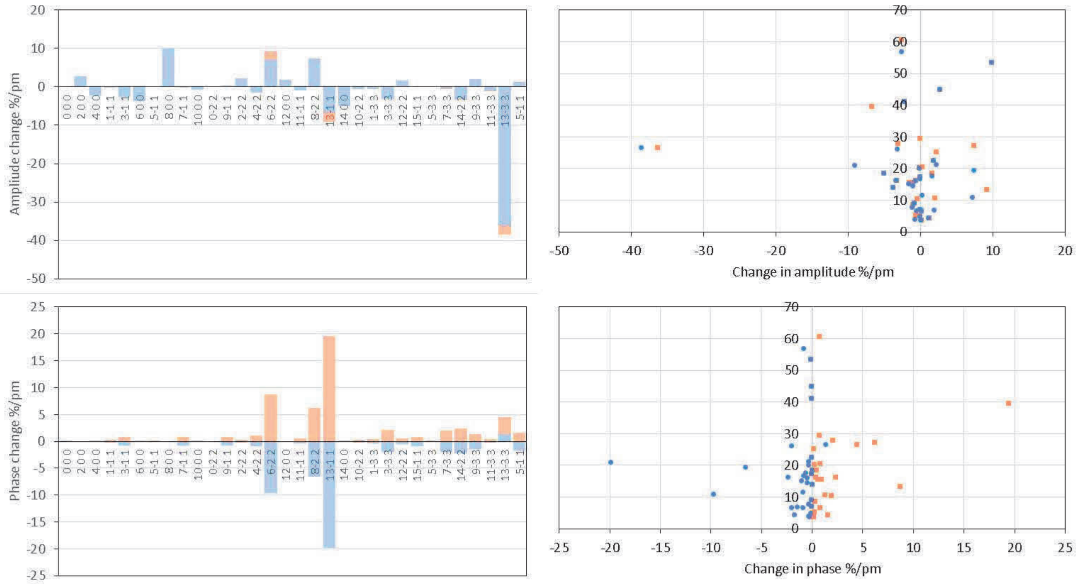

Fig. S37. Left: changes in δF_{ij} amplitude (top) and phase (bottom)of the 54 reflections used in room temperature refinement of the RT $A2_1am$ $Ca_3Mn_2O_7$ structure for a shift of the O3 z coordinate of 1 pm. Right: scatter plot relating the change in amplitude and phases from the plots on the left to the change in normalised intensity Δ_{ij}. Bijvoet reflections are colour coded orange and blue.

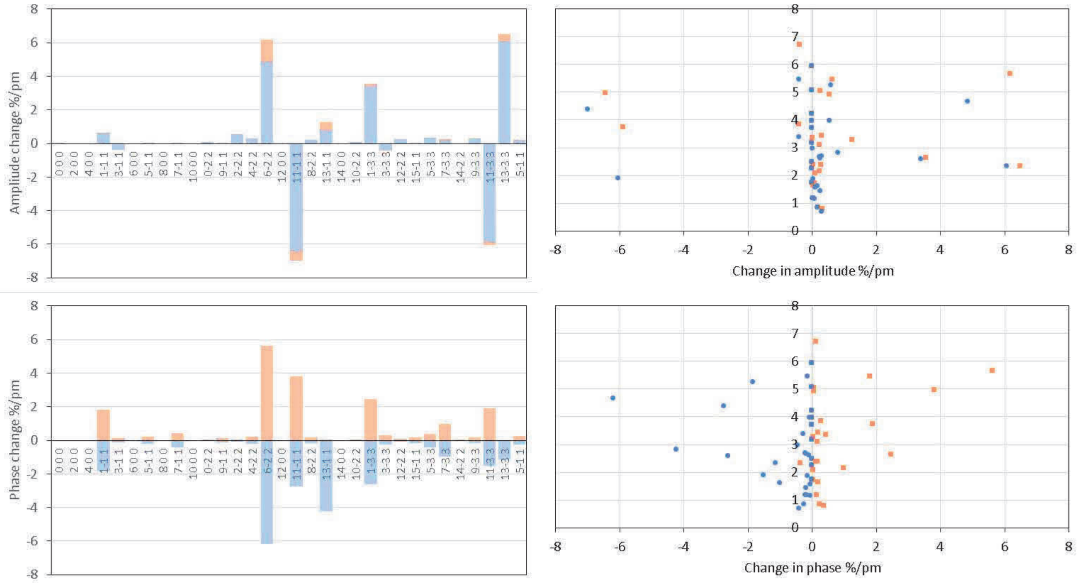

Fig. S38. Left: changes in δF_{ij} amplitude (top) and phase (bottom)of the 54 reflections used in room temperature refinement of the RT $A2_1am$ $Ca_3Mn_2O_7$ structure for a shift of the O3 y coordinate of 1 pm. Right: scatter plot relating the change in amplitude and phases from the plots on the left to the change in normalised intensity Δ_{ij}. Bijvoet reflections are colour coded orange and blue.

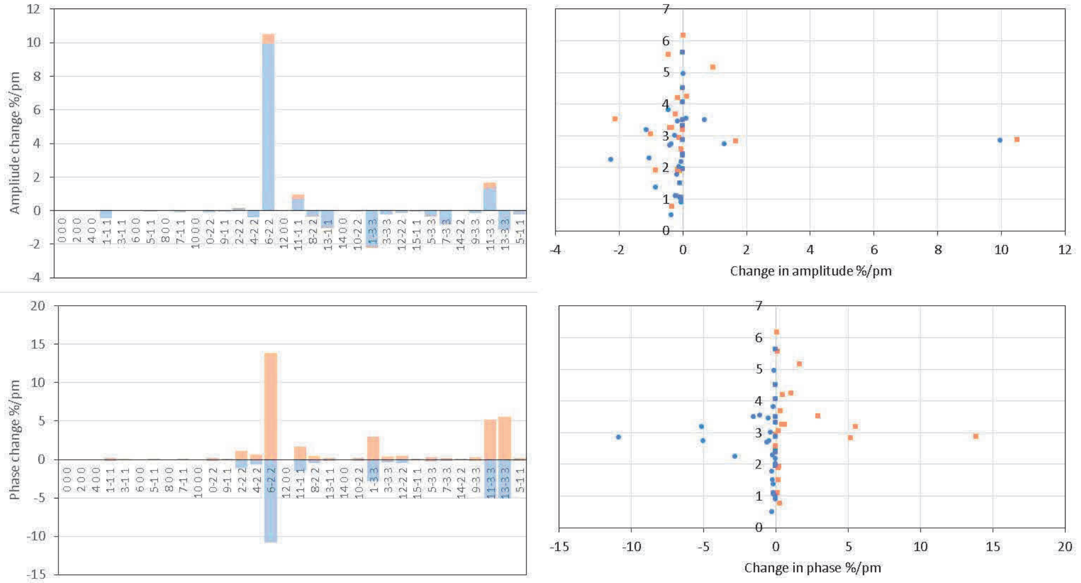

Fig. S39. Left: changes in δF_{ij} amplitude (top) and phase (bottom)of the 54 reflections used in room temperature refinement of the RT $A2_1am$ $Ca_3Mn_2O_7$ structure for a shift of the O3 x coordinate of 1 pm. Right: scatter plot relating the change in amplitude and phases from the plots on the left to the change in normalised intensity Δ_{ij}. Bijvoet reflections are colour coded orange and blue.

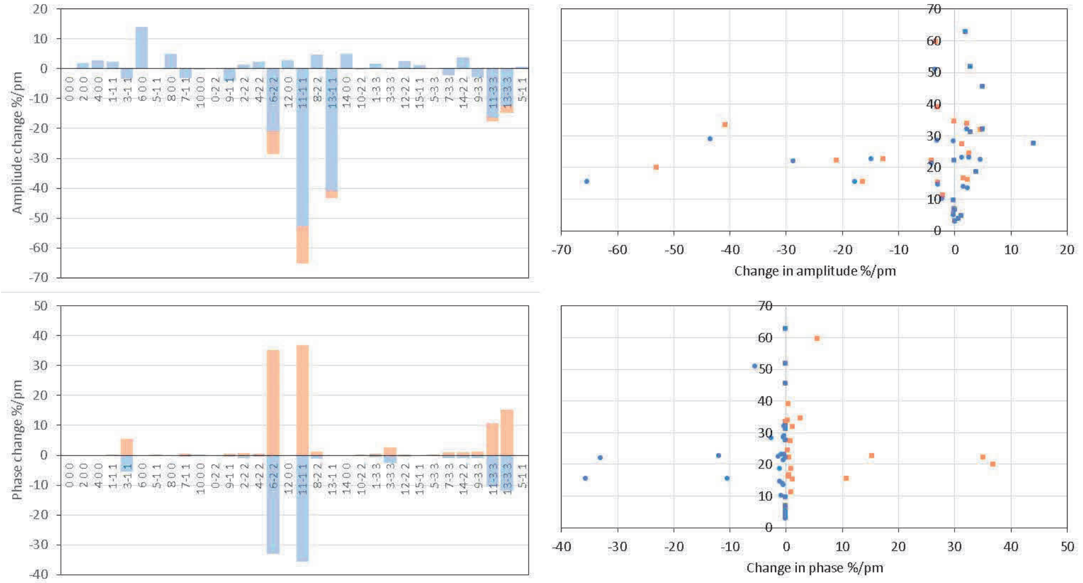

Fig. S40. Left: changes in δF_{ij} amplitude (top) and phase (bottom)of the 54 reflections used in room temperature refinement of the RT $A2_1am$ $Ca_3Mn_2O_7$ structure for a shift of the O4 z coordinate of 1 pm. Right: scatter plot relating the change in amplitude and phases from the plots on the left to the change in normalised intensity Δ_{ij}. Bijvoet reflections are colour coded orange and blue.

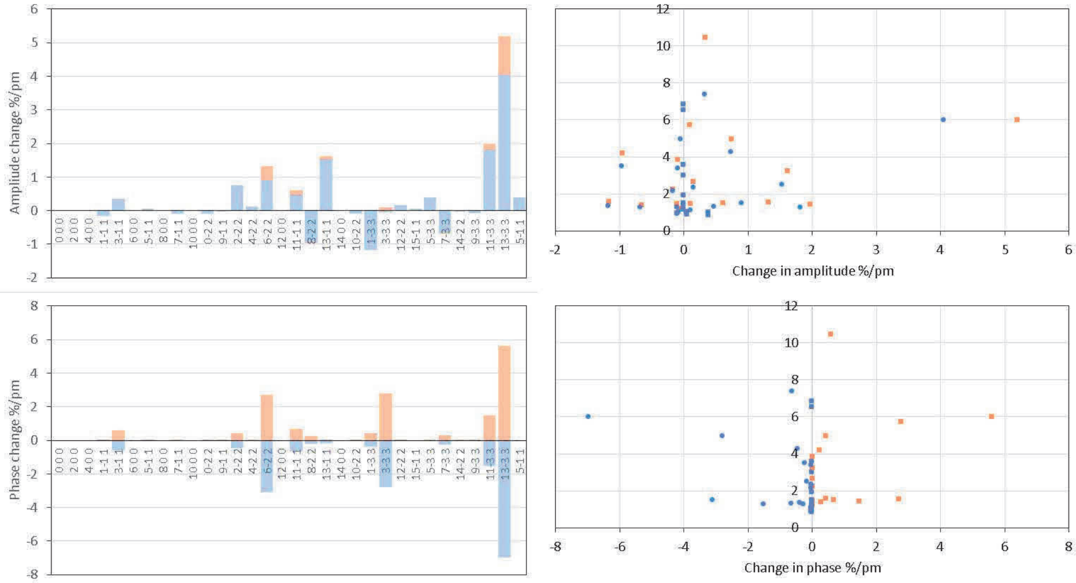

Fig. S41. Left: changes in δF_{ij} amplitude (top) and phase (bottom)of the 54 reflections used in room temperature refinement of the RT $A2_1am$ $Ca_3Mn_2O_7$ structure for a shift of the O4 y coordinate of 1 pm. Right: scatter plot relating the change in amplitude and phases from the plots on the left to the change in normalised intensity Δ_{ij}. Bijvoet reflections are colour coded orange and blue.

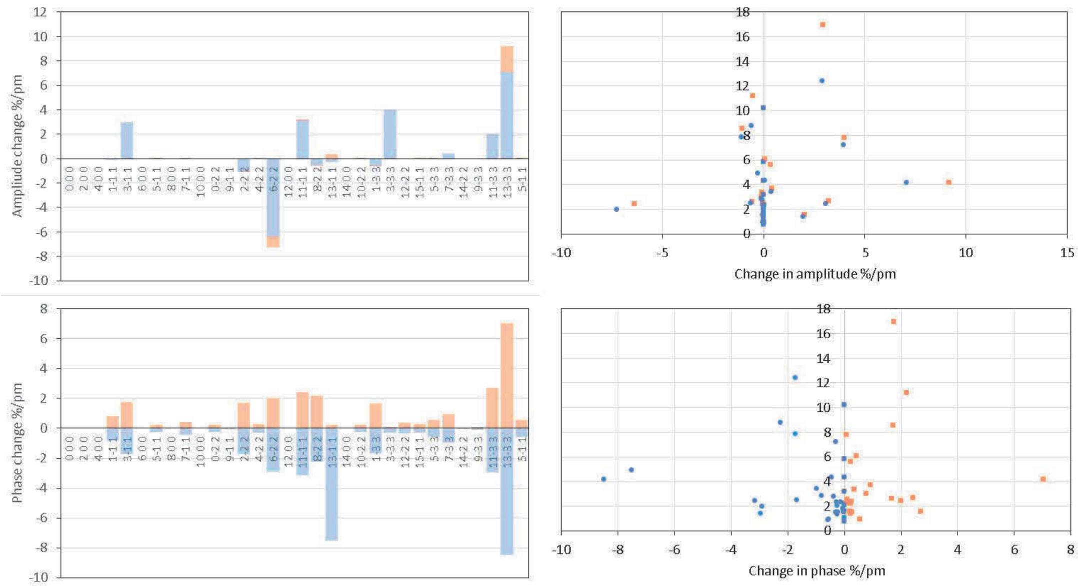

Fig. S42. Left: changes in δF_{ij} amplitude (top) and phase (bottom)of the 54 reflections used in room temperature refinement of the RT $A2_1am$ $Ca_3Mn_2O_7$ structure for a shift of the O4 x coordinate of 1 pm. Right: scatter plot relating the change in amplitude and phases from the plots on the left to the change in normalised intensity Δ_{ij}. Bijvoet reflections are colour coded orange and blue.
